# Egret Swarm Optimization Algorithm: An Evolutionary Computation Approach for Model Free Optimization

**DOI:** 10.3390/biomimetics7040144

**Published:** 2022-09-27

**Authors:** Zuyan Chen, Adam Francis, Shuai Li, Bolin Liao, Dunhui Xiao, Tran Thu Ha, Jianfeng Li, Lei Ding, Xinwei Cao

**Affiliations:** 1 College of Engineering, Swansea University, Swansea SA1 3UA, UK; 2 College of Computer Science and Engineering, Jishou University, Jishou 416000, China; 3 School of Mathematics, Tongji University, Shanghai 200092, China; 4 Institute of Mechanics, Vietnam Academy of Science and Technology, Hanoi 000084, Vietnam; 5 School of Business, Jiangnan University, Wuxi 214122, China

**Keywords:** metaheuristic algorithm, swarm intelligence, egret swarm optimization algorithm, constrained optimization

## Abstract

A novel meta-heuristic algorithm named Egret Swarm Optimization Algorithm (ESOA) is proposed in this paper, which is inspired by two egret species’ hunting behavior (Great Egret and Snowy Egret). ESOA consists of three primary components: a sit-and-wait strategy, aggressive strategy as well as discriminant conditions. The learnable sit-and-wait strategy guides the egret to the most probable solution by applying a pseudo gradient estimator. The aggressive strategy uses random wandering and encirclement mechanisms to allow for optimal solution exploration. The discriminant model is utilized to balance the two strategies. The proposed approach provides a parallel framework and a strategy for parameter learning through historical information that can be adapted to most scenarios and has well stability. The performance of ESOA on 36 benchmark functions as well as 3 engineering problems are compared with Particle Swarm Optimization (PSO), Genetic Algorithm (GA), Differential Evolution (DE), Grey Wolf Optimizer (GWO), and Harris Hawks Optimization (HHO). The result proves the superior effectiveness and robustness of ESOA. ESOA acquires the winner in all unimodal functions and reaches statistic scores all above 9.9, while the scores are better in complex functions as 10.96 and 11.92.

## 1. Introduction

General engineering applications involving manipulator control, path planning, and fault diagnosis can be described as optimization problems. Since these problems are almost non-convex, conventional gradient approaches are difficult to apply and frequently result in local optima [1]. For this reason, meta-heuristic algorithms are being increasingly utilized to solve such problems as they are able to find a sufficiently good solution, whilst not relying on gradient information.

**Figure 1:**
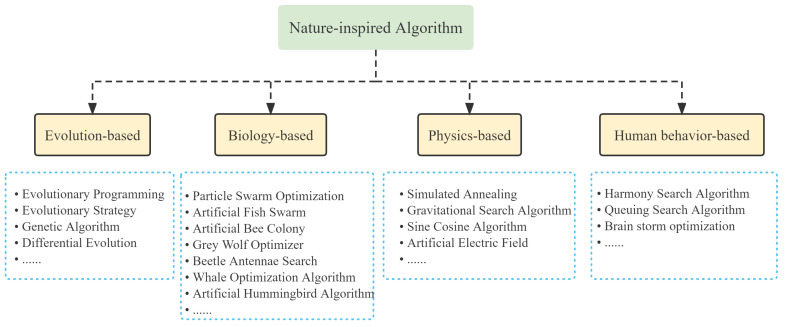
The taxonomy of existing meta-heuristics algorithm.

Meta-heuristic algorithms imitate natural phenomena through simulating animal and environmental behaviours [2]. As shown in Figure 1, these algorithms are broadly inspired by four concepts: the species evolution, the biological behavior, the human behavior as well as the physical principles [3,4].

Evolution-based algorithms generate various solution spaces by mimicking the natural evolution of a species. Potential solutions are considered as members of a population, which evolve over time towards better solutions through a series of cross-mutation and Survival of the fittest. The Darwinian evolution-inspired Genetic Algorithm (GA) has remarkable global search capabilities and has been applied in a variety of disciplines [5]. Comparable to GA, Differential Evolution (DE) has also shown to be able to adapt to a number of optimization problems [6]. In addition, evolutionary strategies [7] and evolutionary programming [8] are among some of the well-known algorithms in this classification. Physics-based approaches apply the physical law as a way to achieve an optimal solution. Common examples include: Simulated Annealing (SA) [9], Gravitational Search Algorithm (GSA) [10], Black Hole Algorithm (BH) [11], and Multi-Verse Optimizer (MVO) [12]. Human behavior-based algorithms simulate the evolution of human society or human intelligence. Examples include the Harmony Search Algorithm (HSA) [13], Queuing Search Algorithm (QSA) [14], as well as the Brain Storm Optimization Algorithm (BSO) [15].

Biologically inspired algorithms mimic behaviours like hunting, pathfinding, growth, and aggregation in order to solve numerical optimization problems. A well known example of this is Particle Swarm Optimization (PSO), a swarm intelligence algorithm inspired by bird flocking behavior [16]. PSO leverages information exchange among individuals in a population to develop the population’s motion from disorder to order, resulting in the location of an optimal solution. Alternatively, ref. [17] was inspired by the behaviour of ant colonies, proposing the Ant Colony Optimization (ACO) algorithm. In ACO, a feasible solution to the optimization problem is represented in terms of the ant pathways, where a greater number of pheromone is deposited on shorter paths. The concentration of pheromone collecting on the shorter pathways steadily rises over time, which causes the ant colony to focus on the optimal path due to the influence of positive feedback.

With the widespread application of PSO and ACO in areas such as robot control, route planning, artificial intelligence, and combinatorial optimization, meta-heuristic algorithms have enabled a plethora of excellent research. Authors in [18] presented Grey Wolf Optimizer (GWO) based on the hierarchy and hunting mechanism of grey wolves. In [19], authors applied GWO to restructure a maximum power extraction model for a photovoltaic system under a partial shading situation. GWO has also been utilized in non-linear servo systems to tune the Takagi-Sugeno parameters of the proportional-integral-fuzzy controller [20]. Paper [21] introduced the Sparrow Search Algorithm (SSA), inspired by the collective foraging and anti-predation behaviours of sparrows. Authors in [22] suggested a novel incremental generation model based on a chaotic Sparrow Search Algorithm to handle large-scale data regression and classification challenges. An integrated optimization model for dynamic reconfiguration of active distribution networks was built with a multi-objective SSA by [23]. Authors in [24] constructed a tiny but efficient meta-heuristic algorithm named Beetle Antennae Search Algorithm (BAS), through modelling the beetle’s predatory behavior. Paper [25] integrated BAS with a recurrent neural network to create a novel robot control framework for redundant robotic manipulator trajectory planning and obstacle avoidance. BAS is applied in [26] to optimize the initial parameters of a convolutional neural network for medical imaging diagnosis, resulting in high accuracy and a short period of tuning time.

In recent years, there has been a proliferation of swarm-based algorithms for various situations due to the massive adoption of swarm intelligence in engineering applications [27,28,29,30,31,32,33,34,35,36]. Authors in [37] proposed a novel meta-heuristic algorithm named Artificial Hummingbird Algorithm (AHA), influenced by the flight skills and hunting strategies of hummingbirds. In addition, paper [38] introduced the African Vultures Optimization Algorithm (AVOA) inspired by vultures’ navigation behavior. Meanwhile, the Starling Maturation Optimizer (SMO) and Orca Predation Algorithm (OPA) were proposed in [39,40] to suit complex optimization problems by mimicking bird migration and orca hunting strategies. Other notable examples include Aptenodytes Forsteri Optimization (AFO) inspired by penguin hugging behaviour [41], Golden Eagle Optimizer (GEO) inspired by golden eagle feeding trails [42], Chameleon Swarm Algorithm (CSA) inspired by Chameleon dynamic hunting routes [43], Red Fox Optimization Algorithm (RFOA) inspired by red fox habits, and Elephant Clan Optimization (ECO) inspired by elephant survival strategies [44,45]. Unlike most other bio-inspired methods, authors in [46] proposed a Quantum-based Avian Navigation Optimizer Algorithm (QANA) that contains a V-echelon topology to disperse information flow and a quantum mutation strategy to enhance search efficiency. Overall, it can be observed that swarm intelligence algorithms have gradually progressed from simply imitating the appearance of animal behavior to modelling the behavior with a deeper understanding of their underlying principles.

Not only that, the evolutionary algorithm performs well on some benchmark functions [47,48,49,50,51,52]. Paper [53] introduced IPOP-CMA-ES utilizing CMA-ES [54] within a restart method with the increasing population size for each restart. Paper [55] integrated IPOP-CMA-ES with an iterative local search as ICMAES-ILS that generated better solution adopted in the rest of evaluations. Authors in [56] developed DRMA that utilizing CMA-ES as the local searcher in GA and dilivering solution space into different parts for global optimization. In addition, there are a series of evolutionary algorithms such as GaAPPADE [57], MVMO14 [58], L-SHADE [59], L-SHADE-ND [60] and SPS-L-SHADE-EIG [61].

Although meta-heuristics algorithms have shown to be well suited to various engineering applications, as Wolpert analyses in [62], there is no near-perfect method that can deal with all optimization problems. To put it another way, if an algorithm is appropriate for one class of optimization problems, it may not be acceptable for another. Furthermore, the search efficiency of an algorithm is inversely related to its computational complexity, and a certain amount of computational consumption needs to be sacrificed in order to enhance search efficiency. However, the No Free Lunch (NFL) theorem has assured that the field has flourished, with new structures and frameworks for meta-heuristics algorithms constantly being developed.

For instance, most metaheuristic algorithms contain just a single strategy, typically a best solution following strategy, and without learnable parameters, which are just deformations built on stochastic optimization. Although this category of algorithms performs well on CEC benchmark functions, they produce disappointing results in praticle application settings because of lacking feedback and parameter-learning mechanism [63,64]. In the field of robotics, the control of a manipulator is a continuous process. If the original metaheuristic algorithm is utilized, the algorithm needs to iterate and converge again at each solution, resulting in a possible discontinuity in the solution space, which affects the control effect [65]. In contrast, the method proposed in this paper includes a learnable tangent surface estimation parameter, which makes it possible to have a base reference to assist in each solution, reducing computational difficulty while ensuring continuity of understanding. Despite the proliferation of studies into meta-heuristic algorithms, the balance between exploitation and exploration has remained a significant topic of research [66,67]. The field requires a framework that balances both, enabling algorithms to be more adaptable and stable in a wider range of situations. This paper proposes a novel meta-heuristic algorithm (Egret Swarm Optimization Algorithm, ESOA) to examine how to improve the balance between the algorithm’s exploration and exploitation. The contributions of Egret Swarm Optimization Algorithm include:

- Proposing a parallel framework to balance exploitation and exploration with excellent performance and stability.
- Introducing a sit-and-wait strategy guided by a pseudo-gradient estimator with learnable parameters.
- Introducing an aggressive strategy controlled by a random wandering and encirclement mechanism.
- Introducing a discriminant condition that are capable of ensembling various strategies.
- Developing a pseudo-gradient estimator referenced to historical data and swarm information.

The rest of the paper is structured as follows: Section 2 depicts the observation of egret migration behavior as well as the development of the ESOA framework and mathematical model. The comparison of performance and efficiency in CEC2005 and CEC2017 between ESOA and other algorithms is demonstrated in Section 3. The result and convergence of two engineering optimization problems utilizing ESOA are discussed in Section 4. Section 5 represents the conclusion of this paper as well as outlines further work.

## 2. Egret Swarm Optimization Algorithm

This section reveals the inspiration of ESOA. Then, the mathematical model of the proposed method is discussed.

### 2.1. Inspiration

The egret is the collective term for four bird species: the Great Egret, the Middle Egret, the Little Egret, and the Yellow-billed Egret, all of which are known for their magnificent white plumage. The majority of egrets inhabit coastal islands, coasts, estuaries, and rivers, as well as lakes, ponds, streams, rice paddies, and marshes near their shores. Egrets are usually observed in pairs, or in small groups, however vast flocks of tens or hundreds can also be spotted [68,69,70]. Maccarone observed that Great Egret fly at an average speed of 9.2 m/s and balance their movements and energy expenditure whilst hunting [71]. Due to the high consumption of energy when flying, the decision to prey typically necessitates a thorough inspection of the trajectory to guarantee that more energy would be obtained through the location of food than what would be expended through flight. Compared to Great Egrets, Snowy Egrets tend to sample more sites, and they will observe and select the location where other birds have already discovered food [72]. Snowy Egrets often adopt a sit-and-wait strategy, a scheme that involves observing the behavior of prey for a period of time and then anticipating their next move in order to hunt with the least energy expenditure [73]. Maccarone indicated in [74] that not only do Snowy Egrets applying the strategy consume less energy, but they are also 50% more efficient at catching prey than other egrets. Although the Great Egret adopt a higher exertion strategy to pursue prey aggressively, they are capable of capturing larger prey since it is rare for large prey to travel through an identical place multiple times [75]. Overall, Great Egrets with an aggressive search strategy balance high energy consumption for potentially greater returns, whereas Snowy Egrets with a sit-and-wait approach, balance lower energy expenditure for smaller but more reliable profits [74].

### 2.2. Mathematical Model and Algorithm

**Figure 2:**
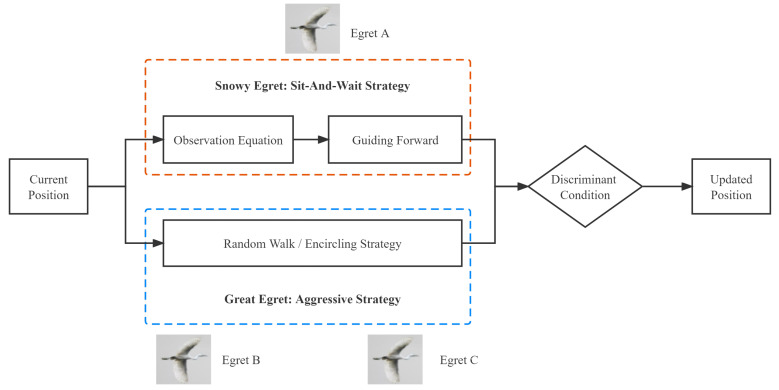
The Framework Of Egret Swarm Optimization Algorithm.

Inspired by the Snowy Egret’s sit-and-wait strategy and the Great Egret’s aggressive strategy, ESOA has combined the advantages of both strategies and constructed a corresponding mathematical model to quantify the behaviors. As shown in the Figure 2, ESOA is a parallel algorithm with three essential components: the sit-and-wait strategy, the aggressive strategy, and the discriminant condition. There are three Egrets in one Egret squad, Egret A applies a guiding forward mechanism while Egret B and Egret C adopt random walk and encircling mechanisms respectively. Each part is detailed below.

**Figure 3:**
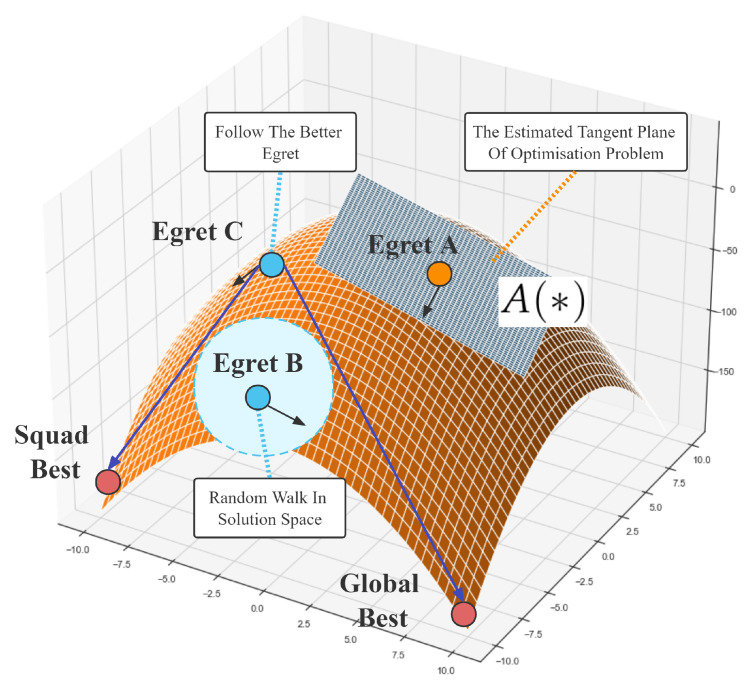
The Detailed Search Behavior of ESOA.

The individual roles and search preferences of the Egret Squad can be seen in Figure 3. Egret A will estimate a descent plane and search based on the gradient of the plane parameters, Egret B performs a global random wander, and Egret C selectively explores based on the location of better egrets. In this way, ESOA will be more balanced in terms of exploitation and exploration and will be capable of performing fast searches for feasible solutions. Unlike gradient descent, ESOA refers to historical information as well as stochasticity in the gradient estimation, meaning it is less likely to fall into the saddle point of the optimization problem. ESOA also differs from other meta-heuristic algorithms by estimating the tangent plane of the optimization problem, enabling a rapid descent to the current optimal point.

#### 2.2.1. Sit-and-Wait Strategy

Observation Equation: Assuming that the position of the *i*-th egret squad is $x_{i}\in R^{n}$, *n* is the dimension of problem, A(*) is the Snowy Egret’s estimate approach of the possible presence of prey in its own current location. $\hat{y}$ is the estimation of prey in current location,

(1) $${\hat{y}}_{i}=A\left(x_{i}\right),$$

then the estimate method could be parameterized as,

(2) $${\hat{y}}_{i}=w_{i}\cdot x_{i},$$

where the $w_{i}\in R^{n}$ is the weight of estimate method. The error $e_{i}$ could be described as,

(3) $$e_{i}={∥{\hat{y}}_{i}-y_{i}∥}^{2}/2.$$

Meanwhile, ${\hat{g}}_{i}\in R^{n}$, the practical gradient of $\omega_{i}$, can be retrieved by taking the partial derivative of $w_{i}$ for the error Equation (3), and its direction is ${\hat{d}}_{i}$.

(4) $$\begin{array}{cc} {\hat{g}}_{i} & =\frac{\partial {\hat{e}}_{i}}{\partial w_{i}}\\ \; & =\frac{\partial {∥{\hat{y}}_{i}-y_{i}∥}^{2}/2}{\partial w_{i}}\\ \; & =\left({\hat{y}}_{i}-y_{i}\right)\cdot x_{i},\\ {\hat{d}}_{i} & ={\hat{g}}_{i}/\left|{\hat{g}}_{i}\right|. \end{array}$$

**Figure 4:**
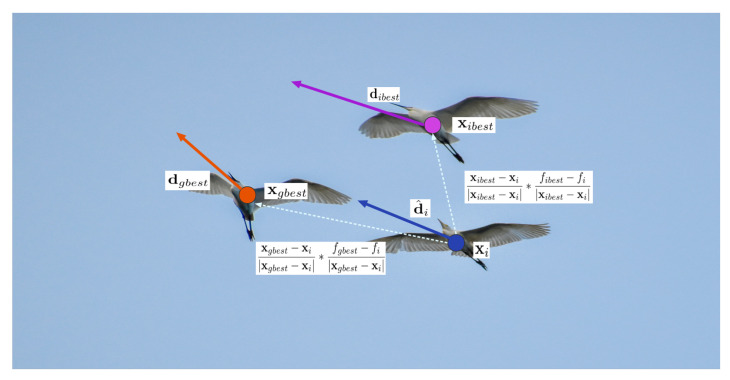
Following behaviour of Egret Swarms as an effective way of gradient estimation.

Figure 4 demonstrates the Egret’s following behavior, where Egrets refer to better Egrets during preying, drawing on their experience of estimating prey behavior and incorporating their own thoughts. $d_{h,i}\in R^{n}$ is the directional correction of the best location of the squad while $d_{g,i}\in R^{n}$ is the the directional correction of the best location of all squad.

(5) $$d_{h,i}=\frac{x_{ibest}-x_{i}}{\left|x_{ibest}-x_{i}\right|}\cdot \frac{f_{ibest}-f_{i}}{\left|x_{ibest}-x_{i}\right|}+d_{ibest}.$$

(6) $$d_{g,i}=\frac{x_{gbest}-x_{i}}{\left|x_{gbest}-x_{i}\right|}\cdot \frac{f_{gbest}-f_{i}}{\left|x_{gbest}-x_{i}\right|}+d_{gbest}.$$

The integrated gradient $g_{i}\in R^{n}$ can be represented as below, and $r_{h}\in \left[0,0.5\right)$, $r_{g}\in \left[0,0.5\right)$:

(7) $$g_{i}=\left(1-r_{h}-r_{g}\right)\cdot {\hat{d}}_{i}+r_{h}\cdot d_{h,i}+r_{g}\cdot d_{g,i}.$$

An adaptive weight update method is applied here [76], $\beta_{1}$ is 0.9 and $\beta_{2}$ is 0.99:

(8) $$\begin{array}{cc} m_{i} & =\beta_{1}\cdot m_{i}+\left(1-\beta_{1}\right)\cdot g_{i},\\ v_{i} & =\beta_{1}\cdot v_{i}+\left(1-\beta_{1}\right)\cdot g_{i}^{2},\\ w_{i} & =w_{i}-m_{i}/\sqrt{v_{i}}. \end{array}$$

According to Egret A’s judgement of the current situation, the next sampling location $x_{a,i}$ can be described as,

(9) $$x_{a,i}=x_{i}+step_{a}\cdot \exp\left(-t/(0.1\cdot t_{max})\right)\cdot hop\cdot g_{i},$$

(10) $$y_{a,i}=f\left(x_{a,i}\right),$$

where *t* and $t_{m}ax$ is the current iteration time and the maximum iteration time, while hop is the gap between the low bound and the up bound of solution space. $step_{a}\in \left(0,1\right]$ is Egret A’s step size factor. $y_{a,i}$ is the fitness of $x_{a,i}$.

#### 2.2.2. Aggressive Strategy

Egret B tends to randomly search prey and its behavior could be depicted as below,

(11) $$x_{b,i}=x_{i}+step_{b}\cdot \tan\left(r_{b,i}\right)\cdot hop/\left(1+t\right),$$

(12) $$y_{b,i}=f\left(x_{b,i}\right),$$

where $r_{b,i}$ is a random number in (−π/2,π/2), $x_{b,i}$ is Egret B’s expected next location and $y_{b,i}$ is the fitness.

Egret C prefers to pursue prey aggressively, so the encircling mechanism is used as the update method of its position:

(13) $$\begin{array}{cc} D_{h} & =x_{ibest}-x_{i},\\ D_{g} & =x_{gbest}-x_{i},\\ x_{c,i} & =\left(1-r_{i}-r_{g}\right)\cdot x_{i}+r_{h}\cdot D_{h}+r_{g}\cdot D_{g}, \end{array}$$

(14) $$y_{c,i}=f\left(x_{c,i}\right).$$

$D_{h}$ is the gap matrix between current location and the best position of this Egret squad while $D_{g}$ compares with the best location of all Egret squads. $x_{c,i}$ is the expected location of Egret C. $step_{b}\in \left(0,1\right]$ is Egret B’s step size factor. $r_{h}$ and $r_{g}$ are random numbers in [0,0.5).

#### 2.2.3. Discriminant Condition

After each member of the Egret squad has decided on its plan, the squad selects the optimal option and takes the action together. $x_{s,i}$ is the solution matrix of *i*-th Egret squad:

(15) $$x_{s,i}=\left[x_{a,i}\;x_{b,i}\;x_{c,i}\right],$$

(16) $$y_{s,i}=\left[y_{a,i}\;y_{b,i}\;y_{c,i}\right],$$

(17) $$c_{i}=argmin\left(y_{s,i}\right),$$

(18) $$x_{i}=\left\{\begin{array}{c} x_{s,i}{{|}_{c_{i}}\;if\;y_{s,i}|}_{c_{i}}<y_{i}\;or\;r<0.3,\\ x_{i}\;\;\;else \end{array}\right..$$

If the minimal value of $y_{s,i}$ is better than current fitness $y_{i}$, the Egret squad accepts the choice. Or if the random number r∈(0,1) is less than 0.3, which means there is 30% possibility to accept a worse plan.

### 2.3. Pseudo Code

Based on the discussion above, the pseudo-code of ESOA is constructed as Algorithm 1, which contains two main functions to retrieve the Egret squad’s expected position matrix and a discriminant condition to choose a better scheme. ESOA requires an initial matrix $x^{0}\in R^{P\times N}$ of the *P* size Egret Swarm position as input, while it returns the optimal position $x_{best}$ and fitness $y_{best}$.

We will analyse the computational complexity of each part of ESOA in turn and provide the final results. For sit-and-wait strategy, Equation (4) requires n+1, Equations (5) and (6) need the same 2n+1 while Equation (7) require 3n+1 floating-point operators. Weight updating Equation (8) and position search need 2n+4 as well as n+1 respectively. Then the total operators of sit-and-wait strategy is 11n+9. As for aggressive strategy, random wander Equation (11) and encircling mechanism require both n+1 then in total 2n+2 operators. Discriminant condition need *n* operators. So ESOA requires a total of 14n+11 floating-point operators and then its computational complexity is O(n). Assuming that the population size of ESOA is *k*, the complexity then becomes O(kn).
**Algorithm 1** Egret Swarm Optimization Algorithm **Input:** $x^{0}$: the *P* size Egret Swarm position $\in R^{P\times N}$, $step_{a}$ as the Egret A’s step size factor while $step_{b}$ as the Egret B’s; **Output:** $x_{best}$: Optimal or approximate optimal solution; $y_{best}$: Optimal or approximate optimal fitness;1:**function**Sitandwait(x)2:    Update the integrated gradient g via Equations (4)–(7)3:    Update the weight of observation method ω by Equation (8)4:    Get the expected position $x_{a}$ of Egret A by Equation (9)5:    Retrieve the Egret A’s fitness $y_{a}$6:    **return** $x_{a}$, $y_{a}$7:**end function**8:**function**Aggressive(x)9:      Get the expected position $x_{b}$ of Egret B by Equation (11)10:    Get the expected position $x_{c}$ of Egret C by Equation (13)11:    Retrieve the fitness of Egret B $y_{b}$ and Egret C $y_{c}$12:    **return** $x_{b},x_{c},y_{a},y_{a}$13:**end function**14:**while** $t<t_{max}$**do**15:    $x_{a}^{t},y_{a}^{t}$←Sitandwait($x^{t}$)16:    $x_{b}^{t},x_{c}^{t},y_{b}^{t},y_{c}^{t}$←Aggressive($x^{t}$)17:    Get next position $x^{t+1}$ via Equations (15)–(18)18:**end while**19:**return** $x_{best},y_{best}$

### 2.4. Parameters

**Figure 5:**
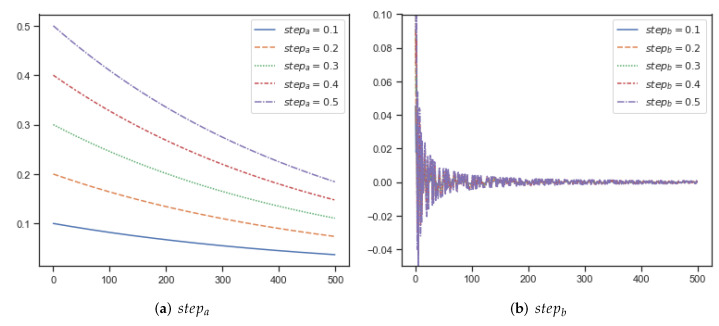
(**a**,**b**) represent the effect of different $step_{a}$ and $step_{b}$ on the step size, respectively.

The two parameters required for ESOA are the step factors $step_{a}\in \left(0,1\right]$ and $step_{b}\in \left(0,1\right]$ for Egret A and Egret B respectively. Larger step coefficients represent more aggressive exploration behavior. Table 1 shows how the parameters required for ESOA compare to other state-of-the-art algorithms. ESOA requires two parameters, which can be easily adjusted to obtain better optimization results when optimizing the problem. In fact, as ESOA’s Equation (8) in the sit-and-wait strategy contains adaptive mechanisms, it is able to respond to different parameter changes by self-adjusting. In the general case, both $step_{a}$ and $step_{b}$ can be set to 0.1 to deal with most problems. Figure 5 presents the effect of various $step_{a}$ and $step_{b}$ on the step size. $step_{a}$ larger a will significantly increase the search step of Egret $step_{a}$ during the original iterations, but will gradually close the gap with a smaller $step_{a}$ after many iterations. In simple applications or unimodal problems, $step_{a}$ can be appropriately tuned up for faster convergence, and in multimodal problems or complex applications, $step_{a}$ is appropriately tuned down for scenarios that require continuous optimization. A larger value of $step_{b}$ means that Egret B will wander randomly in larger steps, suitable for complex scenarios, to help ESOA perform a larger search and jump out of the local optimum solution where possible.

## 3. Experimental Results and Discussion

In this section, the quantified performance of the ESOA algorithm is evaluated by examining 36 optimization functions. The first 7 unimodal functions are typical benchmark optimization problems presented in [8] and the mathematical expressions, dimensions, range of the solution space as well as the best fitness are indicated in Table 2. The final result and partial convergence curves are shown in Table A3 and Table A4 respectively. The remaining 29 test functions introduced in [82] are constructed by summarizing valid features from other benchmark problems, such as cascade, rotation, shift as well as shuffle traps. The overview of these functions are shown in Table 3 and the comparison is indicated in Table A5. All of the experiments are in 30 dimensions, whilst the algorithms used have 50 population sizes and are limited to a maximum of 500 iterations.

**Figure 6:**
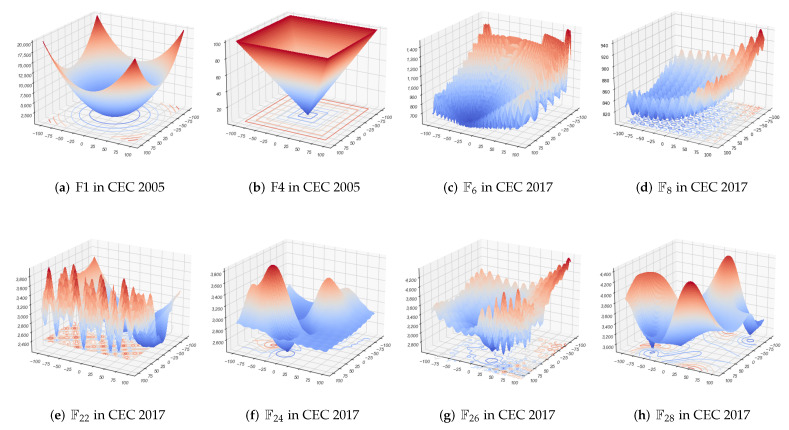
(**a**,**b**) are Unimodal Functions, (**c**,**d**) are Simple Multimodal Functions, (**e**–**h**) are Composition Functions.

Researchers generally classify optimization test functions as Unimodal, Simple Multimodal, Hybrid, and Composition Functions. A 3D visualization of several of these functions are shown in Figure 6. As Unimodal Functions, (a) and (b) are $F_{1}$ as well as $F_{4}$ in CEC 2005, which only have one global minimum value without local optima. (c) and (d), the Simple Multimodal problems, retain numerous local optimal solution traps and multiple peaks to impede the exploration of global optimal search. Hybrid Multimodal functions are a series of problems adding up several different test functions with well-designed weights, and due to the dimension restriction, these are hard to reveal in the 3D graphics. (e), (f), (g) as well as (h) are Composition Functions, the non-linear combination of numerous test functions. These functions are extremely hard to optimize because of the various local traps and spikes, all of which are designed to impede the algorithm’s progress.

ESOA was compared with three traditional algorithms (PSO [16], GA [5], DE [6]) as well as two novel methods (GWO [20], HHO [83]) in the 37 benchmark functions. The numerical results from a maximum of 500 iterations is presented in Table A3 and Table A5. The initial input for each algorithm is a random matrix with 30 dimensions and 50 populations in [−100,100]. The specific variables *w*, $c_{1}$, and $c_{2}$ in PSO are 0.8, 0.5, and 0.5 while the mutation value is 0.001 in GA.

### 3.1. Computational Complexity Analysis

The computational complexity test was performed using a laptop with windows 11, 16 GB of RAM, and an i5-10210U quad core CPU. Table 4 indicates the cost time from 100 runs between ESOA and other algorithms on the CEC05 benchmark functions in 30 dimensions. In general, ESOA is medium in terms of computational complexity, with $F_{4}$ taking the shortest time of all the algorithms and $F_{7}$ the longest.

### 3.2. Evaluation of Exploitation Ability

The exploration and exploitation measurement method utilized in this paper is based on computing the dimension-wise diversity of the meta-heuristic algorithm population in the following way [84,85].

(19) $$\begin{array}{cc} \; & Div_{j}=\frac{1}{n}\sum_{i=1}^{n}median\left(x^{j}\right)-x_{i}^{j},\\ \; & Div=\frac{1}{D}\sum_{j=1}^{D}Div_{j}, \end{array}$$

where $median(x^{j})$ means the median value of algorithm swarm in dimension *j*, whereas $x_{i}^{j}$ is the value of individual *i* in dimension *j*, *n* is the population size. Then Div presents the average value of the whole swarm.

**Figure 7:**
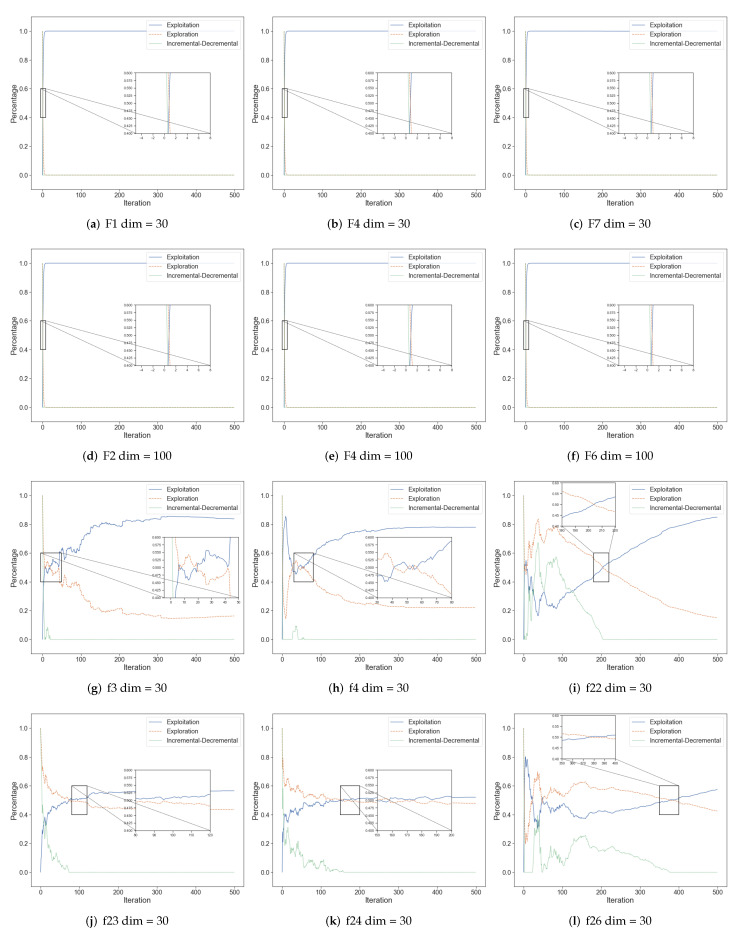
The exploration and exploitation of ESOA in benchmark test.

Moreover, the percentage of exploration and exploitation based on dimension-wise diversity could be calculated as below,

(20) $$\begin{array}{cc} \; & Xpl\%=(\frac{Div}{Div_{max}})\cdot 100,\\ \; & Xpt\%=(\frac{|Div-Div_{max}|}{Div_{max}})\cdot 100, \end{array}$$

where Xpl% is the exploration value while Xpt% indicates the exploitation value. $Div_{max}$ means the maximum diversity along with the whole iteration process. The exploration, exploitation as well as incremental-decremental are shown in Figure 7. Increment means the increasing ability of algorithms’ exploration while decrement presents the contrary. We can find that in most unimodal benchmark functions, due to the fast convergence of ESOA, all agents search the optimal solution swiftly and are clustered together about almost 10 iterations. And in complex functions, ESOA is computed after some iterations and all agents will gradually approach the optimal solution and are distributed around the optimal solution, which is expressed as exploitation gradually overtaking exploration.

The Unimodal Function is utilized to evaluate the convergence speed and exploitation ability of the algorithms, as only one global optimum point is present. As shown in Table 5, ESOA demonstrates outstanding performance from $F_{1}$ to $F_{4}$. ESOA trails GWO and HHO in $F_{5}$ to $F_{7}$, however, the result is considerably superior to PSO, GA, as well as DE. Therefore, the excellent exploitation ability of ESOA is evident here.

### 3.3. Evaluation of Exploration Ability ($F_{3}$–$F_{19}$)

In the MultiModal Functions and Hybrid Functions, there are numerous local optimum positions to impede the algorithm’s progress. The optimization difficulty increases exponentially with rising dimensions, which are useful for evaluating the exploration capability of an algorithm. The result of $F_{3}$–$F_{19}$ shown in Table A5 is clear evidence of the remarkable exploration ability of ESOA. In particular, ESOA has superior performance to the other five algorithms for the average fitness on $F_{18}$. Because of the aggressive strategy component of ESOA, it is capable of overcoming the interference from numerous local optimum points in the exploration of the global solution.

### 3.4. Comprehensive Performance Assessment ($F_{20}$–$F_{29}$)

Composition Functions are a difficult type of test function that require a good balance between exploitation and exploration. They are usually employed to undertake comprehensive evaluations of algorithms. The performance of each algorithm in $F_{20}$–$F_{29}$ is shown in Table A5, the average fitness of ESOA in each test function is extremely competitive when compared to the other listed algorithms. Especially in $F_{22}$, ESOA outperforms the other approaches and reaches 2346 average fitness while the second method (DE) only obtains 3129. In fact, ESOA possesses a sit-and-wait strategy for exploitation as well as an aggressive strategy for exploration. Both features are regulated by a discriminant condition which is fundamental to the performance of the algorithm in such scenarios.

### 3.5. Algorithm Stability

In general, the standard deviation of an algorithm’s outcomes when it is repeatedly applied to a problem can reflect its stability. It can be seen that the standard deviation of ESOA is at the top results in both tables in most situations, and much ahead of the second position in certain test functions. The stability of ESOA is hence proven.

Table A1 and Table A2 are two-sided 5% *t*-test results of ESOA’s performance in CEC05 and CEC17, respectively, against other algorithms. Combining this with Table A3, Table A4 and Table A5, it can be concluded that ESOA outperforms the other algorithms by a wide margin on the benchmark function.

In addition, for the field of evolutionary computation, hypothesis tests with parameters are more difficult to fully satisfy the conditions, so the Wilcoxon non-parametric test has been added to this section [86]. The Wilcoxon test results of ESOA’s performance in CEC05 and CEC17 are indicated in Table A6 and Table A7, which could be an evidence that ESOA demonstrates sufficient superiority.

**Figure 8:**
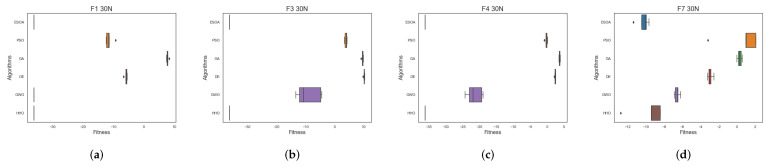
Box plots of the various algorithms’ performances for the CEC05 benchmark function. (**a**) F1 dim = 30, (**b**) F3 dim = 30, (**c**) F4 dim = 30, (**d**) F7 dim = 30.

**Figure 9:**
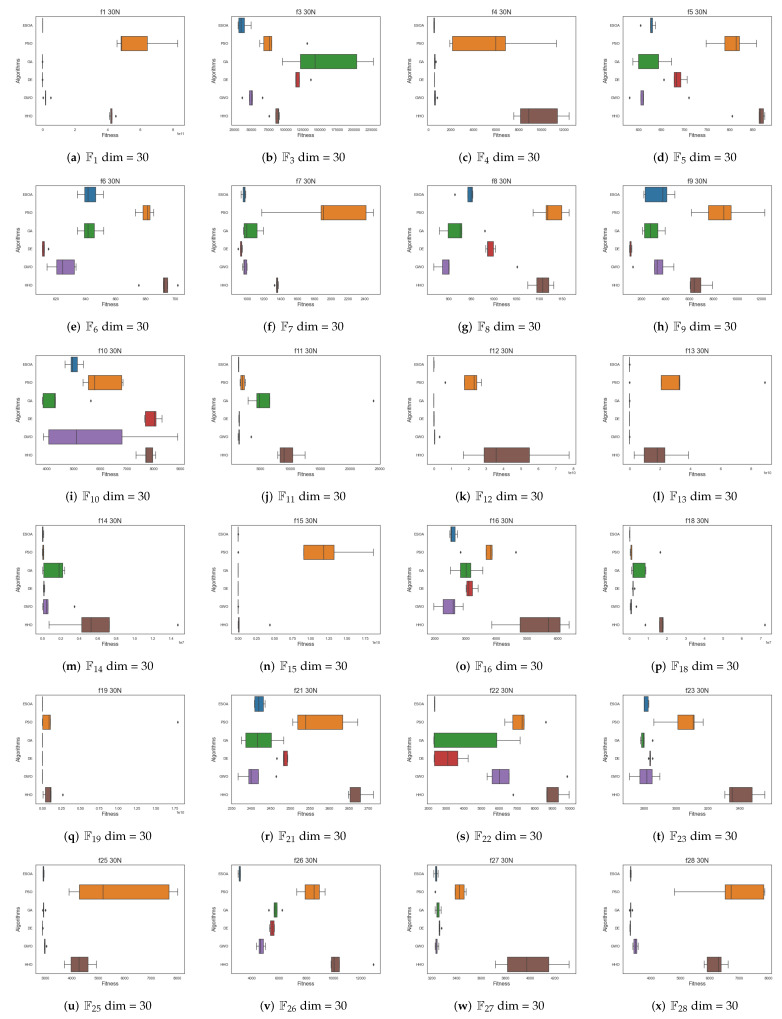
Box Plots of the Various Algorithms’ Performances for the CEC17 Benchmark Function.

Figure 8 and Figure 9 show box plots of the results of multiple algorithms run 30 times on two types of test functions, respectively, where Figure 6 has been ln-processed. The results show that ESOA outperforms the other algorithms in most cases and has a smaller box, indicating a more stable algorithm. In the unimodal test function, ESOA’s boxes are significantly smaller than those of the other algorithms and have narrower boxes. With most complex test functions, e.g., $F_{3}$, $F_{14}$, $F_{22}$ as well as $F_{26}$, ESOA has a significantly smaller box than the other algorithms and its superiority can be clearly seen.

### 3.6. Analysis of Convergence Behavior

**Figure 10:**
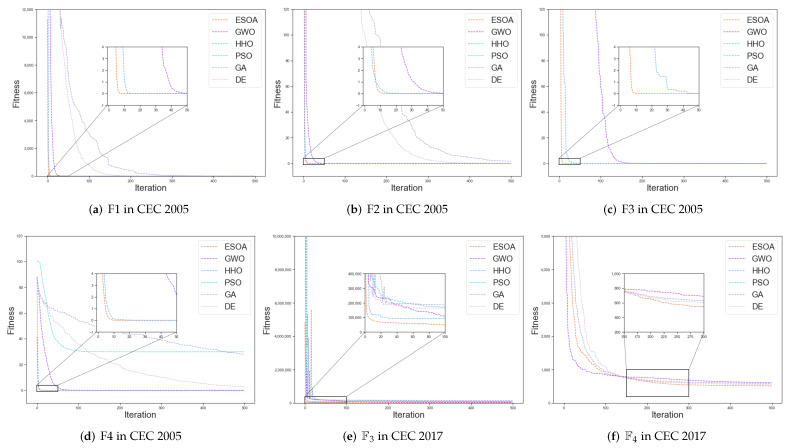

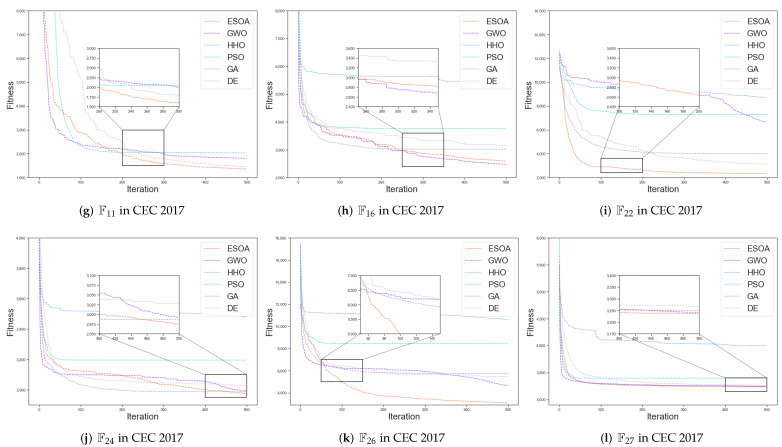
(**a**–**d**) are Unimodal Functions, (**e**,**f**) are Simple Multimodal Functions, (**g**,**h**) are Hybrid Functions, (**i**–**l**) are Composition Functions.

The partial convergence curves of each method are shown in Figure 10. In (a), (b), and (c), the Unimodal Functions, ESOA converges to near the global optimum in less than 10 iterations while PSO, GA as well as DE have yet to uncover the optimal path for fitness descent. The fast convergence in unimodal tasks allows ESOA to be applied to some online optimization problems. In (d), (e), and (f), the Multimodal and Hybrid Functions, after a period of searching the optimization results of ESOA will surpass almost all other algorithms in most cases and would continue to explore afterward. ESOA’s effectiveness in Multimodal problems indicates that it has notable potential to be applied in general engineering applications. In (g), (h) as well as (i), the Composition problems, ESOA’s search, and estimation mechanism allow for continuous optimization in most cases, and ultimately for excellent results. The performance in the Composition Functions is evidence of ESOA’s applicability for use in complex engineering applications.

**Figure 11:**
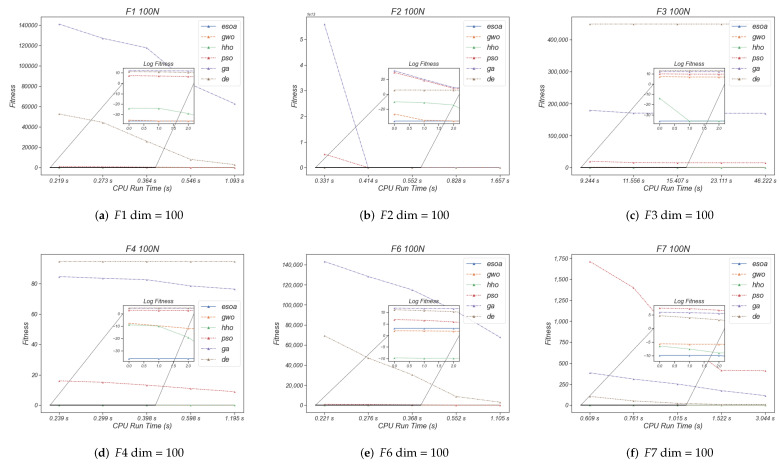

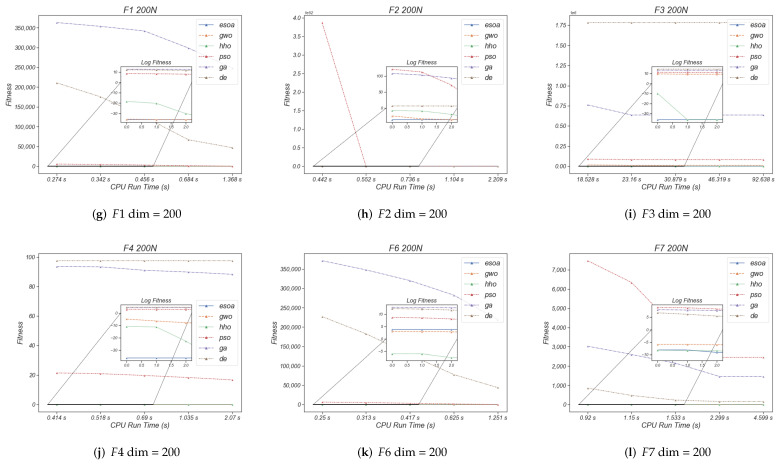
The performance of each algorithm in given CPU run time.

To complete the experiment and to provide more favorable conditions for demonstrating the superiority of ESOA, we have added a supplementary experiment to this section. The experiment uses the 100 and 200 dimensions of the CEC2005 benchmark, with five fixed sets of CPU runtimes present in each experiment. Figure 11 shows the optimal value searched for by each algorithm for a fixed CPU runtime, with a subplot of the logarithm of the fitness value to show its differentiation. The Figure 11 reveals that ESOA always remains the best at most fixed times, for instance (a), (b) and (c) in Figure 11, while the second place is usually taken by GWO or HHO. This experiment further justice to the superiority of ESOA.

### 3.7. Statistical Analysis

In order to clarify the results of the comparison of the algorithms, this section will count the number of winners, losers, scores as well as rankings of each algorithm on the different test functions. The score is calculated as below,

(21) $$S_{i}=\sum_{j=1}^{n}\frac{f_{i}^{j}-F_{min}^{j}}{F_{max}^{j}-F_{min}^{j}}+W_{i},$$

where $S_{i}$ presents the score of *i*-th algorithm, $f_{i}^{j}$ means the optimal value of *i*-th algorithm in *j*-th problem. $F_{min}^{j}$ is the minimal value of all algorithm in *j*-th problem while $F_{max}^{j}$ is the maximal value. And $W_{i}$ is the number of winners of *i*-th algorithm in all problems.

As the CEC 2005 results shown in Table 6 and Table 7, ESOA consistently ranked first among all algorithms in all dimensions, with HHO in second place. ESOA performed particularly well in the 50, 100, and 200 dimensions, all close to 12, pulling away from second place by almost 3 scores. The results of CEC2017 are shown in Table 8, for the simple multimodal problem, ESOA was slightly behind GWO, but the scores were very close, at 8.99378 and 9.01376 respectively. For the hybrid functions and composition functions, ESOA was again the winner and outperformed others. As the data indicates, ESOA is with the capability of fast convergence in simple problems while maintaining excellent generalization and robustness for complex problems. The beneficial properties of ESOA stem from the algorithmic framework’s coordination, where the discrimination conditions effectively balance the exploitation of the sit-and-wait strategy with the exploration of the aggressive strategy.

In order to better reflect the superiority of ESOA, two generic ranking systems, ELO and TrueSkill, are utilized in additional ranking [87]. Table 9, Table 10 and Table 11 indicate the ranking performance of each algorithm on the CEC05 and CEC17 benchmark test sets respectively. In CEC05 benchmark, ESOA reached first place under both ELO rankings and maintained scores above 1450 (ELO applies benchmark score as [1500, 1450, 1400, 1350, 1300, 1250]), and their performance at CEC17 was consistent. ESOA continues to show leadership in the CEC05 test set with scores of 29+ and first place in all CEC17 results under TrueSkill’s evaluation metrics.

In conclusion, this section revealed ESOA’s properties under various test functions. The sit-and-wait strategy in ESOA allows the algorithm to perform fast descent on deterministic surfaces. ESOA’s aggressive strategy ensures that the algorithm is extremely exploratory and does not readily slip into local optima. Therefore, ESOA has shown excellent outcomes in both exploration and exploitation.

## 4. Typical Application

In this section, ESOA is utilized on three practical engineering applications to demonstrate its competitive capabilities on optimization constraint problems. We compare not only ESOA with the original metaheuristic algorithm used in the previous section, but also with some improved variants, such as IWHO [88], QANA [46], L-Shade [59], iL-Shade [89] as well as MPEDE [90]. The results show that although the improved variant of the algorithm is able to achieve better optimization, it still falls short in terms of the adaptability of the constraints, in contrast, ESOA is comfortable with all constraints. Although some methods perform very well in CEC test functions, they may show weak results when applied to real scenarios or applications, which is because each optimisation method has its own aspects that are suitable and is not exhaustive.

In order to simplify the computational procedure, a penalty function is adopted to integrate the inequality constraints into the objective function [91]. The specific form is as below,

(22) $$\hat{f}\left(x\right)=f\left(x\right)+\phi \sum_{j=1}^{p}g_{j}^{2}\left(x\right)sgn\left(g_{j}\left(x\right)\right),$$

(23) $$sgn\left(g_{j}\left(x\right)\right)=\left\{\begin{array}{c} 1,\;ifg_{j}\left(x\right)>0\\ 0,\;ifg_{j}\left(x\right)\leq 0. \end{array}\right.$$

where $\hat{f}\left(x\right)$ is the transformed objective function and ϕ is the penalty parameter while f(x) and $g_{j}\left(x\right)$ is the origin objective function as well as the inequality constraints respectively, j∈[1,2,…,p] and *p* are the number of constraints. $sgn(g_{j}\left(x\right))$ is used to determine whether the independent variable violates a constraint.

### 4.1. Himmelblau’s Nonlinear Optimization Problem

Himmelblau introduced a nonlinear optimization problem as one of the famous benchmark problems for meta-heuristic algorithms [92]. The problem is described below,

$$\begin{array}{cc} \; & Minimize\;\;f\left(x\right)=5.3578547x_{3}^{2}+0.8356891x_{1}x_{5}\\ \; & \;\;\;\;+37.293239x_{1}-40,\;792.141 \end{array}$$

$$\begin{array}{cc} \; & s.t.\;\left\{\begin{array}{c} g_{1}\left(x\right)=85.334407+0.0056858x_{2}x_{5}\\ \;\;+0.0006262x_{1}x_{4}-0.0022053x_{3}x_{5}\\ g_{2}\left(x\right)=80.51249+0.0071317x_{2}x_{5}\\ \;\;+0.0029955x_{1}x_{2}-0.0021813x_{3}^{2}\\ g_{3}\left(x\right)=9.300961+0.0047026x_{3}x_{5}\\ \;\;+0.0012547x_{1}x_{3}-0.0019085x_{3}x_{4}\\ 0\leq g_{1}\left(x\right)\leq 92\\ 90\leq g_{2}\left(x\right)\leq 11\\ 20\leq g_{3}\left(x\right)\leq 25\\ 78\leq x_{1}\leq 102\\ 33\leq x_{2}\leq 45\\ 27\leq x_{3}\leq 45\\ 27\leq x_{4}\leq 45\\ 27\leq x_{5}\leq 45. \end{array}\right. \end{array}$$

For this problem, the ϕ in Equation (22) is set to $10^{100}$, the number of search agents used by each algorithm is set to 10, and the maximum number of iterations is set to 500.

The optimal result is presented in Table 12 while the statistic result from 30 trials for each algorithm is shown in Table 13. PSO is the best performing algorithm in terms of optimal results, with the best result reaching −30,665.5 of variables [78,33,29.9953,45,36.7758]. The second best was achieved by ESOA at −30,664.5 of variables [78,33,29.9984,45,36.7764]. The standard deviation represents the algorithm’s stability, and ESOA, although ranking second, outperforms PSO, GA, DE, as well as HHO. The experimental results demonstrate the engineering feasibility of the proposed method.

### 4.2. Tension/Compression Spring Design

**Figure 12:**
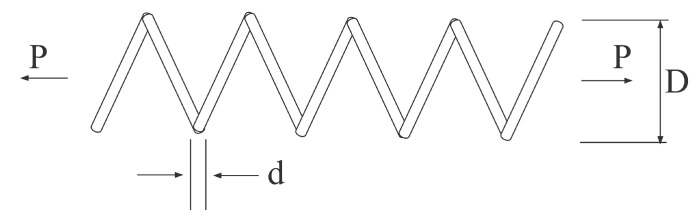
Tension/compression string design problem.

The string design problem is described by Arora and Belegundu for minimizing spring weight under the constraints of minimum deflection, shear stress, and surge frequency [93,94]. Figure 12 illustrates the details of the problem, *P* is the number of active coils, *d* is the wire diameter while *D* represents the mean coil diameter.

The mathematical modeling is given below,

$$\begin{array}{cc} \; & Minimize\;\;f\left(x\right)=\left(x_{3}+2\right)x_{2}x_{1}^{2}\\ \; & s.t.\;\left\{\begin{array}{c} g_{1}\left(x\right)=1-\frac{x_{2}^{3}x_{3}}{71,\;785x_{1}^{4}}\leq 0\\ g_{2}\left(x\right)=\frac{4x_{2}^{2}-x_{1}x_{2}}{12,\;566(x_{2}x_{1}^{3}-x_{1}^{4})}+\frac{1}{5108x_{1}^{2}}-1\leq 0\\ g_{3}\left(x\right)=1-\frac{140.45x_{1}}{x_{2}^{2}x_{3}}\leq 0\\ g_{4}\left(x\right)=\frac{x_{1}+x_{2}}{1.5}-1\leq 0. \end{array}\right. \end{array}$$

For this problem, the ϕ in Equation (22) is set to $10^{5}$, the number of search agents used by each algorithm is again set to 10, and the maximum number of iterations is set to 500.

Table 14 and Table 15 show the optimal and statistic results from 30 independent runs for the six algorithms respectively. The average fitness of DE achieves the best result at 0.0127371, whilst ESOA achieves the second best result at 0.0127839. The standard deviation of ESOA outperforms other algorithms, which demonstrates ESOA’s exceptional stability. The optimal result of ESOA was 0.0127434 of variables [0.05,0.317168,14.0715].

### 4.3. Three-Bar Truss Design

**Figure 13:**
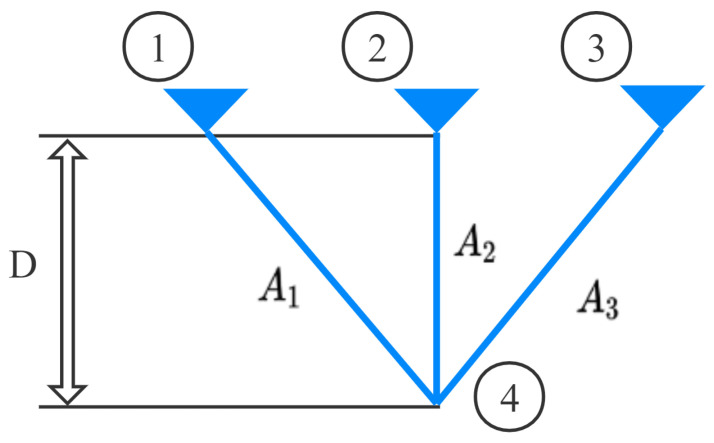
Three-Bar Truss Design Problem.

Three-bar truss design was introduced in [95], whose objective is to optimize the weight under the constraints of stress, deflection, and buckling. Figure 13 presents the structure of three bar truss and the parameters to be optimized. The specific mathematical formula is as below,

$$\begin{array}{cc} \; & Minimize\;\;f\left(x\right)=\left(2\sqrt{2}x_{1}+x_{2}\right)\cdot l\\ \; & s.t.\;\left\{\begin{array}{c} g_{1}\left(x\right)=\frac{\sqrt{2}x_{1}+x_{2}}{\sqrt{2}x_{1}^{2}+2x_{1}x_{2}}P-\sigma \leq 0\\ g_{2}\left(x\right)=\frac{x_{2}}{\sqrt{2}x_{1}^{2}+2x_{1}x_{2}}P-\sigma \leq 0\\ g_{3}\left(x\right)=\frac{1}{\sqrt{2}x_{2}+x_{1}}P-\sigma \leq 0\\ 0\leq x_{1},x_{2}\leq 1\\ l=100\\ P=2\\ \sigma =2 \end{array}\right. \end{array}$$

For this problem, the ϕ in Equation (22) is set to $10^{16}$, the number of search agents used by each algorithm is again set to 10, and the maximum number of iterations is set to 500.

Table 16 indicates ESOA, PSO, DE as well as HHO reach the optimal result of this problem. The optimal result achieved by ESOA is 263.896 with variables [0.788838,0.407793]. Table 17 presents the statistic result of each algorithm and provides sufficient proof for the excellence of ESOA. The proposed algorithm’s (ESOA) effectiveness and robustness are hence verified.

## 5. Conclusions

This paper introduced a novel meta-heuristic algorithm, the Egret Swarm Optimization Algorithm, which mimics two egret species’ typical hunting behavior (Great Egret and Snowy Egret). ESOA consists of three essential components: Snowy Egret’s sit-and-wait strategy, Great Egret’s aggressive strategy as well as a discriminant condition. The performance of ESOA was compared with 5 other state-of-the-art methods (PSO, GA, DE, GWO, and HHO) on 36 test functions, including Unimodal, Multimodal, Hybrid, and Composition Functions. The results of which demonstrate ESOA’s exploitation, exploration, comprehensive performance, stability as well as convergence behavior. In addition, two practical engineering problem instances demonstrate the excellent performance and robustness of ESOA to typical optimization applications. The code developed in this paper is available at https://github.com/Knightsll/Egret_Swarm_Optimization_Algorithm; https://ww2.mathworks.cn/matlabcentral/fileexchange/115595-egret-swarm-optimization-algorithm-esoa (accessed on 26 September 2022).

To accommodate more applications and optimization scenarios, other mathematical forms of sit-and-wait strategy, aggressive strategy, and discriminant condition in ESOA are currently under development.

## Figures and Tables

**Table 1:** The Comparison of Required Parameters.

Algorithm	Mechanism	Parameters
PSO [16]	Bird Predation	$w,c_{1},c_{2},v_{max},v_{min}$
GA [5]	Mutation, Crossover	Selection Rate, Crossover Rate, Mutation Rate
GSO [77]	Galactic Motion	Subswarm, $EP_{max},c_{1},c_{2},c_{3},c_{4}$
CSA [43]	Hiding Place	Awareness Probability, Flight Length
FFA [78]	Multiswarm	W,Q,KValue,α,β
GWO [18]	Social Hierarchy, Encircling Prey	*a*
WOA [79]	Whale Predation	a,b
JS [80]	Active And Passive Motions	β,γ
ALSO [81]	Balanced Lumping	$r_{1},r_{2}$
ESOA	Predation Strategy	$step_{a},step_{b}$

**Table 2:** Unimodal test function.

Function	Dim	Range	$F_{\min}$
$F_{1}\left(x\right)=\sum_{i=1}^{n}x_{i}^{2}$	30	[−100,100]	0
$F_{2}\left(x\right)=\sum_{i=1}^{n}|x_{i}|+\prod_{i=1}^{n}\left|x_{i}\right|$	30	[−10,10]	0
$F_{3}\left(x\right)=\sum_{i=1}^{n}{\left(\sum_{j=1}^{i}x_{j}\right)}^{2}$	30	[−100,100]	0
$F_{4}\left(x\right)=max_{i}\left\{\right|x_{i}\left|,1\leq i\leq n\right\}$	30	[−100,100]	0
$F_{5}\left(x\right)=\sum_{i=1}^{n-1}\left[100{\left(x_{i+1}-x_{i}^{2}\right)}^{2}+{\left(x_{i}-1\right)}^{2}\right]$	30	[−30,30]	0
$F_{6}\left(x\right)=\sum_{i=1}^{n}\left({\left\lfloor x_{i}+0.5\right\rfloor }^{2}\right)$	30	[−100,100]	0
$F_{7}\left(x\right)=\sum_{i=1}^{n}ix_{i}^{4}+random\left[0,1\right)$	30	[−1.28,1.28]	0

**Table 3:** Summary of the CEC’17 Test Functions.

	No.	Functions $\left(F_{i}\right)$	$F_{\min}$
Unimodal Functions	1	Shifted and Rotated Bent Cigar Function	100
	2	Shifted and Rotated Zakharov Function	200
Simple Multimodal Functions	3	Shifted and Rotated Rosenbrock’s Function	300
	4	Shifted and Rotated Rastrigin’s Function	400
	5	Shifted and Rotated Expanded Scaffer’s F6 Function	500
	6	Shifted and Rotated Lunacek Bi_Rastrigin Function	600
	7	Shifted and Rotated Non-Continuous Rastrigin’s Function	700
	8	Shifted and Rotated Levy Function	800
	9	Shifted and Rotated Schwefel’s Function	900
Hybrid Functions	10	Hybrid Function 1 (N = 3)	1000
	11	Hybrid Function 2 (N = 3)	1100
	12	Hybrid Function 3 (N = 3)	1200
	13	Hybrid Function 4 (N = 4)	1300
	14	Hybrid Function 5 (N = 4)	1400
	15	Hybrid Function 6 (N = 4)	1500
	16	Hybrid Function 6 (N = 5)	1600
	17	Hybrid Function 6 (N = 5)	1700
	18	Hybrid Function 6 (N = 5)	1800
	19	Hybrid Function 6 (N = 6)	1900
Composition Functions	20	Composition Function 1 (N = 3)	2000
	21	Composition Function 2 (N = 3)	2100
	22	Composition Function 3 (N = 4)	2200
	23	Composition Function 4 (N = 4)	2300
	24	Composition Function 5 (N = 5)	2400
	25	Composition Function 6 (N = 5)	2500
	26	Composition Function 7 (N = 6)	2600
	27	Composition Function 8 (N = 6)	2700
	28	Composition Function 9 (N = 3)	2800
	29	Composition Function 10 (N = 3)	2900

**Table 4:** The average cost time of each algorithm for the CEC05 problem, Dimension = 30, Maximum Iterations = 500.

	ESOA	PSO [16]	GA [5]	DE [6]	GWO [18]	HHO [83]
F1	0.743205	0.813564	0.932624	0.69359	0.710619	0.820192
F2	1.3374	1.0858	1.268	0.938598	0.9772	1.1564
F3	4.4884	4.35339	4.5192	4.2196	4.2218	6.98319
F4	0.577956	0.682438	0.95496	0.630826	0.580365	0.809334
F5	1.08654	0.848078	1.10613	0.822341	0.743037	1.07561
F6	0.761223	0.75511	1.01592	0.718751	0.646592	0.899095
F7	1.81767	1.05312	1.3103	1.03578	0.953007	1.32848

**Table 5:** Comparison Of Optimization Results Under The Unimodal Functions, Dimension = 30, Maximum Iterations = 500.

F	ESOA		PSO [16]		GA [5]		DE [6]		GWO [18]		HHO [83]	
	ave	std	ave	std	ave	std	ave	std	ave	std	ave	std
$F_{1}$	0.00	0.00	$1.89\times 10^{4}$	$1.10\times 10^{4}$	8.87	$1.69\times 10^{1}$	$7.94\times 10^{-5}$	$1.99\times 10^{-5}$	$8.85\times 10^{-37}$	$5.70\times 10^{-37}$	$1.09\times 10^{-74}$	$1.89\times 10^{-74}$
$F_{2}$	0.00	0.00	$1.12\times 10^{3}$	$1.61\times 10^{2}$	1.76	$9.19\times 10^{-1}$	$3.25\times 10^{-2}$	$6.06\times 10^{-3}$	$1.64\times 10^{-22}$	$2.84\times 10^{-22}$	$9.03\times 10^{-43}$	$1.56\times 10^{-42}$
$F_{3}$	0.00	0.00	$3.72\times 10^{4}$	$1.10\times 10^{4}$	$1.21\times 10^{4}$	$3.07\times 10^{3}$	$1.88\times 10^{4}$	$3.08\times 10^{3}$	$2.54\times 10^{-22}$	$2.75\times 10^{-22}$	$2.58\times 10^{-39}$	$4.46\times 10^{-39}$
$F_{4}$	0.00	0.00	$2.98\times 10^{1}$	6.32	$2.80\times 10^{1}$	2.79	2.73	$1.82\times 10^{-1}$	$5.16\times 10^{-22}$	$3.86\times 10^{-22}$	$2.58\times 10^{-39}$	$4.46\times 10^{-39}$
$F_{5}$	$2.81\times 10^{1}$	$3.46\times 10^{-1}$	$1.01\times 10^{10}$	$6.31\times 10^{9}$	$2.55\times 10^{3}$	$4.34\times 10^{3}$	$1.75\times 10^{2}$	$4.59\times 10^{1}$	$1.24\times 10^{-21}$	$9.80\times 10^{-22}$	$4.96\times 10^{-38}$	$8.00\times 10^{-38}$
$F_{6}$	5.20	$3.82\times 10^{-1}$	$2.04\times 10^{4}$	$1.24\times 10^{4}$	$6.25\times 10^{-1}$	$8.51\times 10^{-1}$	$1.21\times 10^{-4}$	$3.45\times 10^{-5}$	$1.14\times 10^{-21}$	$1.05\times 10^{-21}$	$5.00\times 10^{-38}$	$7.97\times 10^{-38}$
$F_{7}$	$2.26\times 10^{-5}$	$2.25\times 10^{-5}$	$7.64\times 10^{8}$	$5.48\times 10^{8}$	$3.05\times 10^{1}$	$3.40\times 10^{1}$	$1.79\times 10^{-1}$	$2.47\times 10^{-2}$	$4.00\times 10^{-10}$	$6.93\times 10^{-10}$	$4.75\times 10^{-38}$	$8.11\times 10^{-38}$

**Table 6:** The overall rank in CEC 2005 test functions (a), the bold numbers means the best performance among whole competitors.

	Dim = 30				Dim = 50				Dim = 100			
	Winner	Loser	Score	Rank	Winner	Loser	Score	Rank	Winner	Loser	Score	Rank
ESOA	**4**	0	**10.9997**	**1**	**5**	0	**11.9991**	**1**	**5**	0	**11.9996**	**1**
PSO [16]	0	7	0	6	0	1	5.73968	4	0	1	5.40695	4
GA [5]	0	0	5.73443	5	0	4	1.32583	6	0	4	1.06203	6
DE [6]	0	0	6.40334	4	0	2	4.90238	5	0	2	4.07895	5
GWO [18]	0	0	7	3	0	0	6.99967	3	0	0	6.99396	3
HHO [83]	3	0	10	2	2	0	9	2	2	0	9	2

**Table 7:** The overall rank in CEC 2005 test functions (b), the bold numbers means the best performance among whole competitors.

	Dim = 200				Dim = 500				Dim = 1000			
	Winner	Loser	Score	Rank	Winner	Loser	Score	Rank	Winner	Loser	Score	Rank
ESOA	**5**	0	**11.9998**	**1**	**4**	0	**9.99987**	**1**	**4**	0	**9.99989**	**1**
PSO [16]	0	1	5.77841	4	0	1	4.6879	4	0	1	4.59214	4
GA [5]	0	4	1.01684	6	0	3	1.05412	6	0	3	1.10947	6
DE [6]	0	2	4.44476	5	0	2	2.96067	5	0	2	2.73122	5
GWO [18]	0	0	6.97992	3	0	0	5.95328	3	0	0	5.82731	3
HHO [83]	2	0	9	2	2	0	8	2	2	0	8	2

**Table 8:** The overall rank in CEC 2017 test functions (a), the bold numbers means the best performance among whole competitors.

	Simple Multimodal				Hybrid Functions				Composition Functions			
	Winner	Loser	Score	Rank	Winner	Loser	Score	Rank	Winner	Loser	Score	Rank
ESOA	2	0	8.99378	2	3	0	**10.9602**	**1**	**4**	0	**11.9205**	**1**
PSO [16]	0	3	1.94422	5	0	3	3.85613	5	0	2	2.82345	5
GA [5]	1	1	7.23141	4	**4**	0	10.5838	2	1	0	8.27331	3
DE [6]	**3**	1	8.71516	3	0	0	7.66103	4	2	0	9.07179	2
GWO [18]	2	0	**9.01376**	**1**	1	0	8.74242	3	1	0	7.97473	4
HHO [83]	0	3	1.62757	6	0	5	2.11415	6	0	6	0.674735	6

**Table 9:** The ELO and TrueSkill rank in CEC 2005 test functions (a), the bold numbers means the best performance among whole competitors.

	Dim = 30				Dim = 50				Dim = 100			
	ELO	Rank	TrueSkill	Rank	ELO	Rank	TrueSkill	Rank	ELO	Rank	TrueSkill	Rank
ESOA	**1470.39**	**1**	**31.258**	**1**	**1500.9**	**1**	**33.8623**	**1**	**1493.29**	**1**	**33.2997**	**1**
PSO [16]	1383.44	4	24.7029	4	1345.27	4	21.2705	4	1375.78	4	23.8748	4
GA [5]	1273.48	6	15.5308	6	1288.79	6	17.187	6	1281.13	6	16.3589	6
DE [6]	1308.69	5	20.0568	5	1301.03	5	19.2287	5	1293.41	5	18.6219	5
GWO [18]	1397.08	3	27.9015	3	1389.45	3	27.2946	3	1381.83	3	26.6878	3
HHO [83]	1416.93	2	30.55	2	1424.56	2	31.1569	2	1424.56	2	31.1569	2

**Table 10:** The ELO and TrueSkill rank in CEC 2005 test functions (b), the bold numbers means the best performance among whole competitors.

	Dim = 30				Dim = 50				Dim = 100			
	ELO	Rank	TrueSkill	Rank	ELO	Rank	TrueSkill	Rank	ELO	Rank	TrueSkill	Rank
ESOA	**1478.01**	**1**	31.8648	2	**1455.11**	**1**	29.8231	2	**1485.62**	**1**	**32.4274**	**1**
PSO [16]	1352.88	4	21.8331	4	1352.89	4	21.8773	4	1352.89	4	21.8773	4
GA [5]	1281.13	6	16.3589	6	1281.13	6	16.3589	6	1288.79	6	17.187	6
DE [6]	1308.69	5	20.0568	5	1316.3	5	20.6194	5	1293.41	5	18.6219	5
GWO [18]	1397.08	3	27.9015	3	1404.7	3	28.5083	3	1397.08	3	27.9015	3
HHO [83]	1432.22	2	**31.9849**	1	1439.87	2	**32.813**	1	1432.22	2	31.9849	2

**Table 11:** The ELO and TrueSkill rank in CEC 2017 test functions (a), the bold numbers means the best performance among whole competitors.

	Simple Multimodal				Hybrid Functions				Composition Functions			
	ELO	Rank	TrueSkill	Rank	ELO	Rank	TrueSkill	Rank	ELO	Rank	TrueSkill	Rank
ESOA	**1452.45**	**1**	**28.8727**	**1**	**1477.02**	**1**	**29.3237**	**1**	**1481.63**	**1**	**30.4362**	**1**
PSO [16]	1327.35	5	19.0284	6	1383.93	5	22.0796	5	1343.86	5	19.7913	5
GA [5]	1421.97	2	28.0675	2	1432.86	2	27.7156	2	1399.32	4	25.5226	4
DE [6]	1402.15	3	27.3398	4	1403.81	4	25.7017	4	1414.12	2	27.8698	3
GWO [18]	1391.4	4	27.3977	3	1406.88	3	26.9267	3	1413.57	3	28.4724	2
HHO [83]	1284.68	6	19.294	5	1295.51	6	18.2527	6	1280.83	6	17.9076	6

**Table 12:** The optimal result of various algorithms for Himmelblau problem, the bold numbers means the best performance among whole competitors.

	$x_{1}$	$x_{2}$	$x_{3}$	$x_{4}$	$x_{5}$	$g_{1}$	$g_{2}$	$g_{3}$	$g_{4}$	$g_{5}$	$g_{6}$	Value	Constraints
ESOA	78	33	29.9984	45	36.77	−0.00018	−91.9998	−11.16	−8.841	−4.9988	−0.00120342	−30,664.5	Yes
PSO [16]	78	33	29.9953	45	36.77	0	−92	−11.15	−8.84	−5	0	**−30,665.5**	Yes
GA [5]	78.047	35.02	31.81	44.81	32.57	−0.27373	−91.7263	−10.95	−9.04	−4.98832	−0.0116773	−30,333.1	Yes
DE [6]	78	33.00	30.002	44.97	36.77	−0.00107	−91.9989	−11.15	−8.84	−4.99875	−0.00124893	−30,663.2	Yes
GWO [18]	78.0031	33.00	30.0069	45	36.75	−0.00201	−91.998	−11.15	−8.84	−4.99815	−0.0018509	−30,662.7	Yes
HHO [83]	78	33	32.4546	43.68	31.56	−0.86883	−91.1312	−12.05	−7.94	−5	9.56 × 10^−7^	−30,182.6	Yes
L-Shade [59]	78	33	27	27	27	−1.88843	−90.11157	−13.83258	−6.16742	−8.23715	3.23715	−32,217.4	No
iL-Shade [60]	78	33	27	27	27	−1.88843	−90.11157	−13.83258	−6.16742	−8.23715	3.23715	−32,217.4	No
MPEDE [90]	78	33	27	27	27	−1.88843	−90.11157	−13.83258	−6.16742	−8.23715	3.23715	−32,217.4	No

**Table 13:** The statistical result of various algorithms for the Himmelblau problem, the bold numbers means the best performance among whole competitors.

	*Best*	*Worst*	*Ave*	*Std*	*Time*
ESOA	−30,664.5	−30,422.6	−30,615.4	88.457	0.68607
PSO [16]	**−30,665.5**	−30,186.2	−30509.8	200.628	0.64493
GA [5]	−30,333.1	−29,221.1	−29,853.2	312.106	0.71309
DE [6]	−30,663.2	**−30,655.2**	**−30,658.8**	**2.35697**	0.62209
GWO [18]	−30,662.7	−30,453.7	−30,637.6	61.3822	**0.615**
HHO [83]	−30,182.6	−29,643	−29,902.7	186.764	0.85409

**Table 14:** The optimal result of various algorithms for the Spring problem, the bold numbers means the best performance among whole competitors.

	*d*	*D*	*P*	$g_{1}$	$g_{2}$	$g_{3}$	$g_{4}$	Value	Constraints
ESOA	0.05	0.317168	14.0715	−0.000684592	−0.000637523	−3.96102	−0.755221	0.01274345	Yes
PSO [16]	0.05	0.317425	14.0278	5.57 × 10^−8^	$1.31\times 10^{-7}$	−3.96844	−0.75505	0.01271905	No
GA [5]	0.0534462	0.39517	9.57495	−0.00876173	−0.0108724	−4.02034	−0.700922	0.01306582	Yes
DE [6]	0.0516891	0.356718	11.289	$5.38\times 10^{-8}$	$1.22\times 10^{-7}$	−4.05379	−0.727729	0.01266523	No
GWO [18]	0.0532407	0.394828	9.37671	−0.000610472	−0.000786781	−4.11563	−0.701287	**0.01273248**	Yes
HHO [83]	0.0536234	0.405061	8.93075	$5.28\times 10^{-8}$	$1.22\times 10^{-7}$	−4.13981	−0.69421	0.012731469	No
IWHO [88]	0.0517	0.4155	7.1564	−0.000948687	0.132366	−4.87727	−0.688533	0.0102	No
QANA [46]	0.051926	0.362432	10.961632	$4.23\times 10^{-5}$	$-2.82\times 10^{-5}$	−4.06498	−0.72376	0.01266625	No
L-Shade [59]	0.06899394	0.93343162	2	$8.19\times 10^{-8}$	$1.74\times 10^{-7}$	−4.56081	−0.33172	0.01777	No
iL-Shade [89]	0.05573737	0.46215675	7.01862125	$5.43\times 10^{-8}$	$1.22\times 10^{-7}$	−4.22201	−0.65473	0.01295	No
MPEDE [90]	0.05956062	0.5767404	4.71717282	−0.00173	−0.00087	−4.33136	−0.5758	0.01374	Yes

**Table 15:** The statistical result of various algorithms for the spring problem, the bold numbers means the best performance among whole competitors.

	*Best*	*Worst*	*Ave*	*Std*	*Time*
ESOA	0.0127434	**0.0128516**	**0.0127839**	**0.00003092**	0.59006
PSO [16]	0.0127190	0.030455	0.0149345	0.00520118	0.58293
GA [5]	0.0130658	0.0180691	0.0151508	0.00186	0.623
DE [6]	0.0126652	0.0131926	0.0127371	0.000154639	0.56308
GWO [18]	**0.0127324**	0.0136782	0.0131852	0.000340304	**0.535**
HHO [83]	0.0127314	0.0145846	0.0133872	0.000615518	0.64992

**Table 16:** The optimal result of various algorithms for Three-Bar Truss Design problem, the bold numbers means the best performance among whole competitors.

	$A_{1}$	$A_{2}$	$g_{1}$	$g_{2}$	$g_{3}$	Value	Constraints
ESOA	0.788192	0.409618	$-1.34\times 10^{-6}$	−1.46255	−0.948953	**263.896**	Yes
PSO [16]	0.788763	0.408	$-2.29\times 10^{-11}$	−1.46438	−0.949714	263.896	Yes
GA [5]	0.793214	0.395595	$-2.85\times 10^{-5}$	−1.47859	−0.955608	263.914	Yes
DE [6]	0.788675	0.408248	$7.11\times 10^{-15}$	−1.4641	−0.949596	263.896	No
GWO [18]	0.788853	0.40775	$-2.27\times 10^{-6}$	−1.46467	−0.949833	263.897	Yes
HHO [83]	0.788727	0.408102	$5.77\times 10^{-15}$	−1.46427	−0.949665	263.896	No
IWHO [88]	0.7884	0.4081	0.00070	−1.46391	−0.949229	263.8523	No
QANA [46]	0.788675	0.408248	$5.09\times 10^{-7}$	−1.4641	−0.94959	263.895	No
L-Shade [59]	0.78867514	0.40824829	−0.0	−1.4641	−0.9496	263.896	Yes
iL-Shade [89]	0.78867513	0.40824829	−0.0	−1.4641	−0.9496	263.896	Yes
MPEDE [90]	0.78924889	0.40662803	−0.0	−1.46595	−0.95036	263.896	Yes

**Table 17:** The statistical result of various algorithms for Three-Bar Truss Design problem, the bold numbers means the best performance among whole competitors.

	*Best*	*Worst*	*Ave*	*Std*	*Time*
ESOA	**263.896**	263.948	263.909	0.0146444	0.69
PSO [16]	263.896	263.905	263.897	0.00186868	**0.626**
GA [5]	263.914	264.522	264.084	0.158269	0.73401
DE [6]	263.896	**263.896**	**263.896**	$1.14\times 10^{-13}$	0.656
GWO [18]	263.897	263.921	263.904	0.00564346	0.63508
HHO [83]	263.896	268.296	264.424	0.99462	0.78

## Data Availability

The source code used in this work can be retrieved from Github Link: https://github.com/Knightsll/Egret_Swarm_Optimization_Algorithm; Mathworks Link: https://ww2.mathworks.cn/matlabcentral/fileexchange/115595-egret-swarm-optimization-algorithm-esoa.

## Appendix A

**Table A1:** The T-test Result Between ESOA And Other Algorithm On CEC05 Benchmark Functions.

		ESOA vs. PSO	ESOA vs. GA	ESOA vs. DE	ESOA vs. GWO	ESOA vs. HHO
F1	30	$1.96\times 10^{-1}$	$2.74\times 10^{-4}$	$3.36\times 10^{-4}$	$1.56\times 10^{-1}$	$3.40\times 10^{-1}$
	50	$5.54\times 10^{-3}$	$1.08\times 10^{-7}$	$4.20\times 10^{-4}$	$3.29\times 10^{-1}$	$3.39\times 10^{-1}$
	100	$1.52\times 10^{-4}$	$3.99\times 10^{-10}$	$5.46\times 10^{-6}$	$2.40\times 10^{-1}$	$3.43\times 10^{-1}$
	200	$3.19\times 10^{-8}$	$3.20\times 10^{-11}$	$8.71\times 10^{-8}$	$1.46\times 10^{-2}$	$3.13\times 10^{-1}$
	500	$8.22\times 10^{-9}$	$2.06\times 10^{-11}$	$3.65\times 10^{-10}$	$7.16\times 10^{-2}$	$3.46\times 10^{-1}$
	1000	$4.91\times 10^{-13}$	$9.90\times 10^{-15}$	$4.77\times 10^{-13}$	$1.69\times 10^{-1}$	$3.00\times 10^{-1}$
F2	30	$3.44\times 10^{-1}$	$8.45\times 10^{-8}$	$4.66\times 10^{-4}$	$8.41\times 10^{-3}$	$3.27\times 10^{-1}$
	50	$1.99\times 10^{-2}$	$1.00\times 10^{-9}$	$3.41\times 10^{-3}$	$3.61\times 10^{-2}$	$1.37\times 10^{-1}$
	100	$9.30\times 10^{-6}$	$4.49\times 10^{-10}$	$2.75\times 10^{-9}$	$2.98\times 10^{-5}$	$3.16\times 10^{-1}$
	200	$1.88\times 10^{-5}$	$3.40\times 10^{-1}$	$2.86\times 10^{-10}$	$2.52\times 10^{-2}$	$3.45\times 10^{-1}$
	500	N/A	N/A	N/A	N/A	N/A
	1000	N/A	N/A	N/A	N/A	N/A
F3	30	$1.59\times 10^{-4}$	$2.30\times 10^{-5}$	$2.09\times 10^{-6}$	$1.50\times 10^{-1}$	$3.45\times 10^{-1}$
	50	$1.34\times 10^{-5}$	$9.79\times 10^{-8}$	$1.46\times 10^{-8}$	$1.94\times 10^{-1}$	$3.46\times 10^{-1}$
	100	$3.82\times 10^{-6}$	$5.29\times 10^{-8}$	$9.15\times 10^{-9}$	$7.49\times 10^{-2}$	$3.46\times 10^{-1}$
	200	$6.62\times 10^{-7}$	$3.49\times 10^{-9}$	$1.62\times 10^{-10}$	$5.60\times 10^{-2}$	$3.45\times 10^{-1}$
	500	$1.00\times 10^{-6}$	$5.56\times 10^{-7}$	$2.67\times 10^{-8}$	$2.08\times 10^{-4}$	$3.46\times 10^{-1}$
	1000	$1.70\times 10^{-4}$	$1.15\times 10^{-9}$	$5.79\times 10^{-12}$	$3.84\times 10^{-5}$	$3.46\times 10^{-1}$
F4	30	$2.83\times 10^{-4}$	$5.77\times 10^{-7}$	$5.42\times 10^{-7}$	$1.31\times 10^{-1}$	$1.70\times 10^{-1}$
	50	$3.16\times 10^{-9}$	$6.08\times 10^{-10}$	$4.45\times 10^{-14}$	$1.56\times 10^{-1}$	$3.44\times 10^{-1}$
	100	$7.53\times 10^{-10}$	$1.00\times 10^{-11}$	$7.05\times 10^{-17}$	$5.18\times 10^{-2}$	$1.49\times 10^{-1}$
	200	$2.74\times 10^{-11}$	$1.42\times 10^{-14}$	$8.74\times 10^{-19}$	$2.53\times 10^{-1}$	$3.45\times 10^{-1}$
	500	$1.62\times 10^{-18}$	$2.12\times 10^{-19}$	$1.43\times 10^{-18}$	$1.02\times 10^{-2}$	$3.43\times 10^{-1}$
	1000	$2.35\times 10^{-14}$	$1.99\times 10^{-20}$	$1.25\times 10^{-23}$	$1.54\times 10^{-1}$	$3.38\times 10^{-1}$
F5	30	$3.04\times 10^{-1}$	$5.37\times 10^{-4}$	$1.49\times 10^{-2}$	$2.45\times 10^{-3}$	$4.84\times 10^{-15}$
	50	$3.48\times 10^{-3}$	$8.27\times 10^{-6}$	$2.24\times 10^{-7}$	$1.56\times 10^{-2}$	$3.27\times 10^{-18}$
	100	$2.02\times 10^{-5}$	$7.37\times 10^{-6}$	$9.74\times 10^{-5}$	$2.36\times 10^{-4}$	$6.96\times 10^{-26}$
	200	$9.17\times 10^{-7}$	$1.81\times 10^{-7}$	$8.59\times 10^{-7}$	$8.25\times 10^{-3}$	$2.39\times 10^{-28}$
	500	$8.30\times 10^{-10}$	$1.21\times 10^{-13}$	$8.00\times 10^{-7}$	$3.30\times 10^{-6}$	$4.42\times 10^{-28}$
	1000	$2.83\times 10^{-7}$	$2.94\times 10^{-12}$	$1.16\times 10^{-9}$	$1.98\times 10^{-10}$	$2.11\times 10^{-32}$
F6	30	$8.30\times 10^{-11}$	$1.30\times 10^{-5}$	$8.35\times 10^{-11}$	$4.62\times 10^{-8}$	$8.30\times 10^{-11}$
	50	$2.54\times 10^{-12}$	$4.36\times 10^{-7}$	$2.18\times 10^{-1}$	$9.58\times 10^{-10}$	$2.36\times 10^{-12}$
	100	$1.86\times 10^{-3}$	$7.20\times 10^{-8}$	$5.20\times 10^{-6}$	$5.17\times 10^{-10}$	$5.57\times 10^{-14}$
	200	$3.51\times 10^{-5}$	$2.68\times 10^{-11}$	$6.50\times 10^{-7}$	$2.78\times 10^{-13}$	$2.95\times 10^{-17}$
	500	$9.92\times 10^{-12}$	$3.78\times 10^{-12}$	$8.12\times 10^{-14}$	$1.57\times 10^{-11}$	$6.46\times 10^{-18}$
	1000	$2.94\times 10^{-12}$	$5.14\times 10^{-16}$	$6.98\times 10^{-12}$	$2.94\times 10^{-12}$	$5.02\times 10^{-25}$
F7	30	$2.69\times 10^{-2}$	$8.32\times 10^{-6}$	$4.97\times 10^{-5}$	$5.94\times 10^{-5}$	$9.44\times 10^{-2}$
	50	$1.56\times 10^{-2}$	$1.51\times 10^{-4}$	$2.20\times 10^{-8}$	$4.37\times 10^{-2}$	$7.51\times 10^{-1}$
	100	$6.15\times 10^{-3}$	$4.18\times 10^{-6}$	$4.96\times 10^{-3}$	$1.31\times 10^{-3}$	$2.04\times 10^{-2}$
	200	$1.44\times 10^{-6}$	$7.58\times 10^{-7}$	$5.34\times 10^{-8}$	$1.13\times 10^{-2}$	$5.03\times 10^{-2}$
	500	$1.20\times 10^{-7}$	$1.20\times 10^{-8}$	$5.49\times 10^{-9}$	$2.02\times 10^{-4}$	$1.14\times 10^{-1}$
	1000	$1.12\times 10^{-15}$	$9.85\times 10^{-14}$	$2.03\times 10^{-8}$	$2.02\times 10^{-3}$	$2.19\times 10^{-1}$

**Table A2:** The T-test Result Between ESOA And Other Algorithm On CEC17 Benchmark Functions.

		ESOA vs. PSO	ESOA vs. GA	ESOA vs. DE	ESOA vs. GWO	ESOA vs. HHO
$F_{1}$	30	$3.67\times 10^{-5}$	$5.62\times 10^{-6}$	$5.10\times 10^{-6}$	$2.43\times 10^{-2}$	$3.74\times 10^{-12}$
$F_{2}$	30	$3.00\times 10^{1}$	N/A	N/A	N/A	N/A
$F_{3}$	30	$6.70\times 10^{-3}$	$1.36\times 10^{-3}$	$3.59\times 10^{-7}$	$5.66\times 10^{-2}$	$3.95\times 10^{-6}$
$F_{4}$	30	$1.84\times 10^{-2}$	$1.37\times 10^{-2}$	$1.24\times 10^{-2}$	$5.99\times 10^{-2}$	$1.11\times 10^{-5}$
$F_{5}$	30	$1.24\times 10^{-5}$	$7.94\times 10^{-1}$	$4.51\times 10^{-4}$	$9.45\times 10^{-1}$	$2.22\times 10^{-7}$
$F_{6}$	30	$7.26\times 10^{-6}$	$9.66\times 10^{-1}$	$9.61\times 10^{-6}$	$5.74\times 10^{-3}$	$1.50\times 10^{-5}$
$F_{7}$	30	$2.71\times 10^{-3}$	$1.24\times 10^{-1}$	$2.25\times 10^{-2}$	$4.34\times 10^{-1}$	$1.83\times 10^{-9}$
$F_{8}$	30	$2.57\times 10^{-6}$	$3.17\times 10^{-1}$	$4.07\times 10^{-4}$	$5.53\times 10^{-1}$	$1.08\times 10^{-6}$
$F_{9}$	30	$1.42\times 10^{-3}$	$4.00\times 10^{-1}$	$1.77\times 10^{-3}$	$8.00\times 10^{-1}$	$7.60\times 10^{-4}$
$F_{10}$	30	$1.35\times 10^{-2}$	$1.12\times 10^{-1}$	$1.24\times 10^{-7}$	$4.59\times 10^{-1}$	$2.35\times 10^{-7}$
$F_{11}$	30	$4.22\times 10^{-3}$	$1.02\times 10^{-1}$	$5.09\times 10^{-3}$	$3.09\times 10^{-1}$	$8.77\times 10^{-6}$
$F_{12}$	30	$6.83\times 10^{-4}$	$2.42\times 10^{-3}$	$7.10\times 10^{-1}$	$1.69\times 10^{-1}$	$3.89\times 10^{-3}$
$F_{13}$	30	$4.45\times 10^{-2}$	$8.06\times 10^{-5}$	$1.30\times 10^{-1}$	$9.86\times 10^{-1}$	$1.64\times 10^{-2}$
$F_{14}$	30	$3.80\times 10^{-1}$	$3.71\times 10^{-2}$	$2.80\times 10^{-3}$	$2.09\times 10^{-1}$	$2.47\times 10^{-2}$
$F_{15}$	30	$8.83\times 10^{-3}$	$7.38\times 10^{-3}$	$6.95\times 10^{-3}$	$9.96\times 10^{-1}$	$3.15\times 10^{-1}$
$F_{16}$	30	$3.68\times 10^{-3}$	$4.48\times 10^{-2}$	$1.60\times 10^{-4}$	$5.30\times 10^{-1}$	$3.42\times 10^{-4}$
$F_{17}$	30	N/A	N/A	N/A	N/A	N/A
$F_{18}$	30	$2.51\times 10^{-1}$	$3.69\times 10^{-2}$	$2.13\times 10^{-5}$	$7.57\times 10^{-2}$	$5.29\times 10^{-2}$
$F_{19}$	30	$2.88\times 10^{-1}$	$1.99\times 10^{-3}$	$2.64\times 10^{-1}$	$5.94\times 10^{-1}$	$4.46\times 10^{-2}$
$F_{20}$	30	N/A	N/A	N/A	N/A	N/A
$F_{21}$	30	$1.87\times 10^{-3}$	$9.39\times 10^{-1}$	$3.49\times 10^{-5}$	$4.91\times 10^{-1}$	$4.34\times 10^{-8}$
$F_{22}$	30	$1.47\times 10^{-6}$	$1.55\times 10^{-1}$	$7.08\times 10^{-2}$	$7.70\times 10^{-4}$	$2.22\times 10^{-6}$
$F_{23}$	30	$2.25\times 10^{-3}$	$5.58\times 10^{-1}$	$8.23\times 10^{-3}$	$9.60\times 10^{-1}$	$1.76\times 10^{-6}$
$F_{24}$	30	$3.39\times 10^{-5}$	$5.48\times 10^{-1}$	$9.30\times 10^{-4}$	$5.54\times 10^{-1}$	$1.12\times 10^{-4}$
$F_{25}$	30	$9.93\times 10^{-3}$	$6.24\times 10^{-1}$	$1.63\times 10^{-3}$	$8.30\times 10^{-3}$	$2.24\times 10^{-4}$
$F_{26}$	30	$5.41\times 10^{-7}$	$1.90\times 10^{-7}$	$1.33\times 10^{-9}$	$1.22\times 10^{-6}$	$1.33\times 10^{-6}$
$F_{27}$	30	$7.35\times 10^{-3}$	$2.10\times 10^{-1}$	$2.43\times 10^{-3}$	$5.00\times 10^{-1}$	$1.19\times 10^{-4}$
$F_{28}$	30	$2.77\times 10^{-4}$	$8.84\times 10^{-1}$	$4.03\times 10^{-2}$	$1.54\times 10^{-3}$	$5.86\times 10^{-8}$
$F_{29}$	30	N/A	N/A	N/A	N/A	N/A

**Table A3:** The Detailed Comparison of Various Algorithms on Various Dimensions of CEC05 Benchmark Functions(a).

		ESOA				PSO [16]				GA [5]			
		ave	best	worst	std	ave	best	worst	std	ave	best	worst	std
F1	50	0.00	0.00	0.00	0.00	$3.22\times 10^{-2}$	$1.17\times 10^{-2}$	$5.14\times 10^{-2}$	$1.71\times 10^{-2}$	$9.31\times 10^{3}$	$8.31\times 10^{3}$	$1.13\times 10^{4}$	$1.05\times 10^{3}$
	100	0.00	0.00	0.00	0.00	$1.19\times 10^{1}$	7.48	$1.59\times 10^{1}$	3.58	$6.01\times 10^{4}$	$5.39\times 10^{4}$	$6.34\times 10^{4}$	$3.35\times 10^{3}$
	200	0.00	0.00	0.00	0.00	$2.14\times 10^{2}$	$1.83\times 10^{2}$	$2.48\times 10^{2}$	$2.08\times 10^{1}$	$2.34\times 10^{5}$	$2.26\times 10^{5}$	$2.48\times 10^{5}$	$9.51\times 10^{3}$
	500	0.00	0.00	0.00	0.00	$4.41\times 10^{3}$	$4.13\times 10^{3}$	$5.11\times 10^{3}$	$3.60\times 10^{2}$	$9.52\times 10^{5}$	$8.86\times 10^{5}$	$9.95\times 10^{5}$	$3.66\times 10^{4}$
	1000	0.00	0.00	0.00	0.00	$3.14\times 10^{4}$	$2.99\times 10^{4}$	$3.20\times 10^{4}$	$7.56\times 10^{2}$	$2.38\times 10^{6}$	$2.33\times 10^{6}$	$2.42\times 10^{6}$	$3.52\times 10^{4}$
F2	50	0.00	0.00	0.00	0.00	$1.48\times 10^{1}$	$3.54\times 10^{-1}$	$3.08\times 10^{1}$	$1.02\times 10^{1}$	$6.79\times 10^{1}$	$6.20\times 10^{1}$	$7.47\times 10^{1}$	4.25
	100	0.00	0.00	0.00	0.00	$1.02\times 10^{2}$	$7.85\times 10^{1}$	$1.39\times 10^{2}$	$2.07\times 10^{1}$	$2.14\times 10^{2}$	$1.93\times 10^{2}$	$2.30\times 10^{2}$	$1.21\times 10^{1}$
	200	0.00	0.00	0.00	0.00	$3.85\times 10^{2}$	$2.78\times 10^{2}$	$5.13\times 10^{2}$	$8.59\times 10^{1}$	$4.56\times 10^{33}$	$3.02\times 10^{24}$	$2.25\times 10^{34}$	$9.00\times 10^{33}$
	500	N/A	N/A	N/A	N/A	$2.58\times 10^{40}$	$1.73\times 10^{3}$	$1.29\times 10^{41}$	$5.17\times 10^{40}$	N/A	N/A	N/A	N/A
	1000	N/A	N/A	N/A	N/A	$1.33\times 10^{3}$	$1.26\times 10^{3}$	$1.37\times 10^{3}$	$3.83\times 10^{1}$	N/A	N/A	N/A	N/A
F3	50	0.00	0.00	0.00	0.00	$1.03\times 10^{3}$	$7.13\times 10^{2}$	$1.30\times 10^{3}$	$2.19\times 10^{2}$	$4.84\times 10^{4}$	$4.01\times 10^{4}$	$5.71\times 10^{4}$	$5.41\times 10^{3}$
	100	0.00	0.00	0.00	0.00	$1.22\times 10^{4}$	$9.63\times 10^{3}$	$1.52\times 10^{4}$	$2.20\times 10^{3}$	$1.59\times 10^{5}$	$1.43\times 10^{5}$	$1.87\times 10^{5}$	$1.64\times 10^{4}$
	200	0.00	0.00	0.00	0.00	$7.73\times 10^{4}$	$6.22\times 10^{4}$	$9.23\times 10^{4}$	$1.10\times 10^{4}$	$6.69\times 10^{5}$	$6.28\times 10^{5}$	$7.56\times 10^{5}$	$4.90\times 10^{4}$
	500	0.00	0.00	0.00	0.00	$4.28\times 10^{5}$	$3.43\times 10^{5}$	$5.37\times 10^{5}$	$6.46\times 10^{4}$	$3.57\times 10^{6}$	$2.87\times 10^{6}$	$4.21\times 10^{6}$	$5.00\times 10^{5}$
	1000	0.00	0.00	0.00	0.00	$1.97\times 10^{6}$	$1.34\times 10^{6}$	$2.94\times 10^{6}$	$6.00\times 10^{5}$	$1.38\times 10^{7}$	$1.27\times 10^{7}$	$1.50\times 10^{7}$	$8.83\times 10^{5}$
F4	50	0.00	0.00	0.00	0.00	2.84	2.49	3.14	$2.05\times 10^{-1}$	$5.70\times 10^{1}$	$5.22\times 10^{1}$	$6.20\times 10^{1}$	3.35
	100	0.00	0.00	0.00	0.00	8.52	7.75	9.19	$5.14\times 10^{-1}$	$7.43\times 10^{1}$	$7.05\times 10^{1}$	$7.79\times 10^{1}$	2.61
	200	0.00	0.00	0.00	0.00	$1.71\times 10^{1}$	$1.59\times 10^{1}$	$1.77\times 10^{1}$	$6.81\times 10^{-1}$	$8.72\times 10^{1}$	$8.47\times 10^{1}$	$8.83\times 10^{1}$	1.34
	500	0.00	0.00	0.00	0.00	$2.53\times 10^{1}$	$2.52\times 10^{1}$	$2.55\times 10^{1}$	$1.26\times 10^{-1}$	$9.52\times 10^{1}$	$9.48\times 10^{1}$	$9.58\times 10^{1}$	$3.66\times 10^{-1}$
	1000	0.00	0.00	0.00	0.00	$3.09\times 10^{1}$	$2.99\times 10^{1}$	$3.13\times 10^{1}$	$5.10\times 10^{-1}$	$9.75\times 10^{1}$	$9.71\times 10^{1}$	$9.78\times 10^{1}$	$2.79\times 10^{-1}$
F5	50	$4.82\times 10^{1}$	$4.79\times 10^{1}$	$4.85\times 10^{1}$	$2.60\times 10^{-1}$	$2.98\times 10^{2}$	$1.79\times 10^{2}$	$4.55\times 10^{2}$	$1.22\times 10^{2}$	$1.17\times 10^{7}$	$7.47\times 10^{6}$	$1.46\times 10^{7}$	$2.34\times 10^{6}$
	100	$9.84\times 10^{1}$	$9.83\times 10^{1}$	$9.85\times 10^{1}$	$5.77\times 10^{-2}$	$7.26\times 10^{3}$	$5.64\times 10^{3}$	$1.02\times 10^{4}$	$1.61\times 10^{3}$	$9.76\times 10^{7}$	$7.39\times 10^{7}$	$1.30\times 10^{8}$	$1.91\times 10^{7}$
	200	$1.98\times 10^{2}$	$1.98\times 10^{2}$	$1.98\times 10^{2}$	$5.26\times 10^{-2}$	$3.69\times 10^{5}$	$2.80\times 10^{5}$	$4.53\times 10^{5}$	$5.51\times 10^{4}$	$5.91\times 10^{8}$	$5.22\times 10^{8}$	$7.19\times 10^{8}$	$7.15\times 10^{7}$
	500	$4.98\times 10^{2}$	$4.98\times 10^{2}$	$4.98\times 10^{2}$	$1.00\times 10^{-1}$	$1.90\times 10^{7}$	$1.70\times 10^{7}$	$2.03\times 10^{7}$	$1.16\times 10^{6}$	$3.25\times 10^{9}$	$3.18\times 10^{9}$	$3.33\times 10^{9}$	$6.59\times 10^{7}$
	1000	$9.98\times 10^{2}$	$9.98\times 10^{2}$	$9.98\times 10^{2}$	$6.05\times 10^{-2}$	$2.22\times 10^{8}$	$1.91\times 10^{8}$	$2.61\times 10^{8}$	$2.84\times 10^{7}$	$9.20\times 10^{9}$	$8.90\times 10^{9}$	$9.62\times 10^{9}$	$2.77\times 10^{8}$
F6	50	$1.07\times 10^{1}$	$1.03\times 10^{1}$	$1.11\times 10^{1}$	$3.16\times 10^{-1}$	$4.50\times 10^{-2}$	$2.39\times 10^{-2}$	$1.08\times 10^{-1}$	$3.22\times 10^{-2}$	$1.27\times 10^{4}$	$1.07\times 10^{4}$	$1.52\times 10^{4}$	$1.72\times 10^{3}$
	100	$2.31\times 10^{1}$	$2.25\times 10^{1}$	$2.36\times 10^{1}$	$4.24\times 10^{-1}$	$1.05\times 10^{1}$	4.15	$1.89\times 10^{1}$	5.51	$6.26\times 10^{4}$	$5.48\times 10^{4}$	$7.30\times 10^{4}$	$6.73\times 10^{3}$
	200	$4.84\times 10^{1}$	$4.80\times 10^{1}$	$4.89\times 10^{1}$	$3.46\times 10^{-1}$	$2.09\times 10^{2}$	$1.46\times 10^{2}$	$2.51\times 10^{2}$	$3.91\times 10^{1}$	$2.26\times 10^{5}$	$2.19\times 10^{5}$	$2.43\times 10^{5}$	$8.97\times 10^{3}$
	500	$1.23\times 10^{2}$	$1.22\times 10^{2}$	$1.24\times 10^{2}$	$7.27\times 10^{-1}$	$4.18\times 10^{3}$	$4.07\times 10^{3}$	$4.46\times 10^{3}$	$1.42\times 10^{2}$	$9.29\times 10^{5}$	$8.92\times 10^{5}$	$9.58\times 10^{5}$	$2.88\times 10^{4}$
	1000	$2.48\times 10^{2}$	$2.48\times 10^{2}$	$2.48\times 10^{2}$	$1.89\times 10^{-1}$	$3.09\times 10^{4}$	$2.92\times 10^{4}$	$3.17\times 10^{4}$	$9.23\times 10^{2}$	$2.32\times 10^{6}$	$2.29\times 10^{6}$	$2.35\times 10^{6}$	$2.36\times 10^{4}$
F7	50	$3.23\times 10^{-5}$	$8.69\times 10^{-6}$	$6.41\times 10^{-5}$	$1.93\times 10^{-5}$	$2.24\times 10^{1}$	6.00	$4.62\times 10^{1}$	$1.47\times 10^{1}$	$1.26\times 10^{1}$	7.68	$1.88\times 10^{1}$	3.76
	100	$2.49\times 10^{-5}$	$1.20\times 10^{-5}$	$5.01\times 10^{-5}$	$1.34\times 10^{-5}$	$1.99\times 10^{2}$	$1.08\times 10^{2}$	$4.10\times 10^{2}$	$1.08\times 10^{2}$	$1.64\times 10^{2}$	$1.14\times 10^{2}$	$1.91\times 10^{2}$	$2.99\times 10^{1}$
	200	$1.91\times 10^{-5}$	$2.88\times 10^{-6}$	$4.90\times 10^{-5}$	$1.61\times 10^{-5}$	$2.45\times 10^{3}$	$1.94\times 10^{3}$	$2.96\times 10^{3}$	$3.88\times 10^{2}$	$1.74\times 10^{3}$	$1.46\times 10^{3}$	$2.20\times 10^{3}$	$2.53\times 10^{2}$
	500	$2.42\times 10^{-5}$	$5.43\times 10^{-6}$	$5.10\times 10^{-5}$	$1.59\times 10^{-5}$	$4.12\times 10^{4}$	$3.66\times 10^{4}$	$4.95\times 10^{4}$	$4.73\times 10^{3}$	$2.65\times 10^{4}$	$2.42\times 10^{4}$	$3.02\times 10^{4}$	$2.27\times 10^{3}$
	1000	$2.21\times 10^{-5}$	$4.51\times 10^{-6}$	$4.16\times 10^{-5}$	$1.22\times 10^{-5}$	$2.39\times 10^{5}$	$2.34\times 10^{5}$	$2.42\times 10^{5}$	$2.69\times 10^{3}$	$1.41\times 10^{5}$	$1.38\times 10^{5}$	$1.46\times 10^{5}$	$2.78\times 10^{3}$

**Table A4:** The Detailed Comparison Of Various Algorithms On Various Dimensions Of CEC05 Benchmark Functions(b).

		DE [6]				GWO [18]				HHO [83]			
		ave	best	worst	std	ave	best	worst	std	ave	best	worst	std
F1	50	$1.72\times 10^{1}$	9.23	$2.65\times 10^{1}$	5.98	$1.36\times 10^{-43}$	$7.04\times 10^{-46}$	$6.63\times 10^{-43}$	$2.63\times 10^{-43}$	$5.35\times 10^{-76}$	$3.87\times 10^{-86}$	$2.64\times 10^{-75}$	$1.05\times 10^{-75}$
	100	$3.35\times 10^{3}$	$2.85\times 10^{3}$	$4.58\times 10^{3}$	$6.33\times 10^{2}$	$1.37\times 10^{-40}$	$6.06\times 10^{-42}$	$5.70\times 10^{-40}$	$2.17\times 10^{-40}$	$6.46\times 10^{-74}$	$1.16\times 10^{-77}$	$3.21\times 10^{-73}$	$1.28\times 10^{-73}$
	200	$4.44\times 10^{4}$	$3.81\times 10^{4}$	$5.24\times 10^{4}$	$4.89\times 10^{3}$	$2.66\times 10^{-39}$	$2.64\times 10^{-40}$	$4.90\times 10^{-39}$	$1.71\times 10^{-39}$	$1.77\times 10^{-73}$	$3.81\times 10^{-89}$	$8.38\times 10^{-73}$	$3.31\times 10^{-73}$
	500	$3.32\times 10^{5}$	$3.12\times 10^{5}$	$3.62\times 10^{5}$	$1.83\times 10^{4}$	$9.00\times 10^{-37}$	$1.61\times 10^{-38}$	$2.35\times 10^{-36}$	$8.68\times 10^{-37}$	$5.92\times 10^{-71}$	$6.29\times 10^{-80}$	$2.96\times 10^{-70}$	$1.18\times 10^{-70}$
	1000	$9.89\times 10^{5}$	$9.61\times 10^{5}$	$1.03\times 10^{6}$	$2.37\times 10^{4}$	$7.99\times 10^{-36}$	$1.28\times 10^{-37}$	$2.89\times 10^{-35}$	$1.05\times 10^{-35}$	$1.73\times 10^{-69}$	$1.61\times 10^{-79}$	$7.96\times 10^{-69}$	$3.12\times 10^{-69}$
F2	50	5.86	2.43	$1.02\times 10^{1}$	2.85	$6.47\times 10^{-28}$	$1.69\times 10^{-28}$	$1.56\times 10^{-27}$	$5.14\times 10^{-28}$	$3.71\times 10^{-37}$	$1.93\times 10^{-39}$	$1.13\times 10^{-36}$	$4.49\times 10^{-37}$
	100	$1.64\times 10^{2}$	$1.42\times 10^{2}$	$1.75\times 10^{2}$	$1.16\times 10^{1}$	$6.78\times 10^{-26}$	$5.15\times 10^{-26}$	$9.05\times 10^{-26}$	$1.60\times 10^{-26}$	$7.65\times 10^{-39}$	$3.14\times 10^{-43}$	$3.62\times 10^{-38}$	$1.43\times 10^{-38}$
	200	$4.61\times 10^{2}$	$4.34\times 10^{2}$	$4.98\times 10^{2}$	$2.47\times 10^{1}$	$3.59\times 10^{-24}$	$6.36\times 10^{-25}$	$7.94\times 10^{-24}$	$2.62\times 10^{-24}$	$1.33\times 10^{-36}$	$4.44\times 10^{-42}$	$6.65\times 10^{-36}$	$2.66\times 10^{-36}$
	500	$1.44\times 10^{3}$	$1.37\times 10^{3}$	$1.50\times 10^{3}$	$5.27\times 10^{1}$	$3.02\times 10^{-23}$	$2.07\times 10^{-23}$	$3.84\times 10^{-23}$	$6.20\times 10^{-24}$	$1.00\times 10^{-38}$	$1.25\times 10^{-42}$	$4.96\times 10^{-38}$	$1.97\times 10^{-38}$
	1000	N/A	N/A	N/A	N/A	$5.10\times 10^{-23}$	$3.14\times 10^{-23}$	$7.40\times 10^{-23}$	$1.37\times 10^{-23}$	$3.14\times 10^{-36}$	$2.63\times 10^{-46}$	$1.55\times 10^{-35}$	$6.21\times 10^{-36}$
F3	50	$9.96\times 10^{4}$	$8.80\times 10^{4}$	$1.13\times 10^{5}$	$8.75\times 10^{3}$	$1.49\times 10^{1}$	$7.67\times 10^{-1}$	$5.56\times 10^{1}$	$2.11\times 10^{1}$	$3.82\times 10^{-49}$	$2.22\times 10^{-74}$	$1.91\times 10^{-48}$	$7.65\times 10^{-49}$
	100	$4.75\times 10^{5}$	$4.27\times 10^{5}$	$5.38\times 10^{5}$	$3.93\times 10^{4}$	$2.83\times 10^{3}$	$2.90\times 10^{2}$	$8.03\times 10^{3}$	$2.77\times 10^{3}$	$1.49\times 10^{-52}$	$8.99\times 10^{-61}$	$7.48\times 10^{-52}$	$2.99\times 10^{-52}$
	200	$1.76\times 10^{6}$	$1.64\times 10^{6}$	$1.87\times 10^{6}$	$8.77\times 10^{4}$	$3.52\times 10^{4}$	$1.20\times 10^{4}$	$9.74\times 10^{4}$	$3.15\times 10^{4}$	$2.95\times 10^{-50}$	$1.61\times 10^{-60}$	$1.47\times 10^{-49}$	$5.89\times 10^{-50}$
	500	$1.05\times 10^{7}$	$8.72\times 10^{6}$	$1.15\times 10^{7}$	$1.00\times 10^{6}$	$4.49\times 10^{5}$	$2.48\times 10^{5}$	$6.42\times 10^{5}$	$1.40\times 10^{5}$	$1.78\times 10^{-45}$	$2.04\times 10^{-57}$	$8.92\times 10^{-45}$	$3.57\times 10^{-45}$
	1000	$4.31\times 10^{7}$	$4.12\times 10^{7}$	$4.54\times 10^{7}$	$1.41\times 10^{6}$	$4.04\times 10^{6}$	$2.77\times 10^{6}$	$5.44\times 10^{6}$	$9.94\times 10^{5}$	$1.57\times 10^{-19}$	$2.63\times 10^{-60}$	$7.87\times 10^{-19}$	$3.15\times 10^{-19}$
F4	50	$9.11\times 10^{1}$	$8.85\times 10^{1}$	$9.35\times 10^{1}$	1.62	$8.10\times 10^{-8}$	$9.67\times 10^{-9}$	$2.83\times 10^{-7}$	$1.03\times 10^{-7}$	$5.25\times 10^{-36}$	$1.20\times 10^{-41}$	$2.62\times 10^{-35}$	$1.04\times 10^{-35}$
	100	$9.56\times 10^{1}$	$9.46\times 10^{1}$	$9.68\times 10^{1}$	$7.61\times 10^{-1}$	$2.21\times 10^{-6}$	$1.22\times 10^{-7}$	$5.87\times 10^{-6}$	$1.94\times 10^{-6}$	$4.69\times 10^{-38}$	$1.96\times 10^{-40}$	$1.54\times 10^{-37}$	$5.89\times 10^{-38}$
	200	$9.76\times 10^{1}$	$9.71\times 10^{1}$	$9.84\times 10^{1}$	$4.48\times 10^{-1}$	$8.10\times 10^{-4}$	$1.42\times 10^{-5}$	$3.43\times 10^{-3}$	$1.31\times 10^{-3}$	$9.14\times 10^{-34}$	$1.20\times 10^{-43}$	$4.55\times 10^{-33}$	$1.82\times 10^{-33}$
	500	$9.88\times 10^{1}$	$9.80\times 10^{1}$	$9.93\times 10^{1}$	$4.83\times 10^{-1}$	$4.07\times 10^{-1}$	$8.65\times 10^{-2}$	$6.77\times 10^{-1}$	$2.44\times 10^{-1}$	$1.19\times 10^{-37}$	$8.57\times 10^{-44}$	$5.93\times 10^{-37}$	$2.37\times 10^{-37}$
	1000	$9.95\times 10^{1}$	$9.93\times 10^{1}$	$9.97\times 10^{1}$	$1.13\times 10^{-1}$	7.84	1.37	$2.75\times 10^{1}$	9.97	$8.51\times 10^{-38}$	$9.46\times 10^{-42}$	$4.19\times 10^{-37}$	$1.67\times 10^{-37}$
F5	50	$3.65\times 10^{3}$	$2.82\times 10^{3}$	$4.09\times 10^{3}$	$4.48\times 10^{2}$	$4.67\times 10^{1}$	$4.55\times 10^{1}$	$4.83\times 10^{1}$	$9.89\times 10^{-1}$	$1.94\times 10^{-2}$	$2.66\times 10^{-3}$	$6.93\times 10^{-2}$	$2.50\times 10^{-2}$
	100	$2.76\times 10^{6}$	$1.24\times 10^{6}$	$3.38\times 10^{6}$	$7.74\times 10^{5}$	$9.76\times 10^{1}$	$9.73\times 10^{1}$	$9.81\times 10^{1}$	$2.66\times 10^{-1}$	$1.01\times 10^{-2}$	$2.34\times 10^{-3}$	$2.94\times 10^{-2}$	$1.02\times 10^{-2}$
	200	$5.41\times 10^{7}$	$4.13\times 10^{7}$	$6.31\times 10^{7}$	$8.00\times 10^{6}$	$1.96\times 10^{2}$	$1.95\times 10^{2}$	$1.97\times 10^{2}$	$9.89\times 10^{-1}$	$3.27\times 10^{-2}$	$3.09\times 10^{-3}$	$7.82\times 10^{-2}$	$2.47\times 10^{-2}$
	500	$6.95\times 10^{8}$	$5.87\times 10^{8}$	$8.68\times 10^{8}$	$1.01\times 10^{8}$	$4.95\times 10^{2}$	$4.95\times 10^{2}$	$4.96\times 10^{2}$	$4.04\times 10^{-1}$	$1.01\times 10^{-1}$	$8.89\times 10^{-3}$	$3.33\times 10^{-1}$	$1.21\times 10^{-1}$
	1000	$2.44\times 10^{9}$	$2.17\times 10^{9}$	$2.63\times 10^{9}$	$1.56\times 10^{8}$	$9.94\times 10^{2}$	$9.94\times 10^{2}$	$9.94\times 10^{2}$	$1.76\times 10^{-1}$	$8.92\times 10^{-2}$	$2.61\times 10^{-2}$	$2.16\times 10^{-1}$	$6.81\times 10^{-2}$
F6	50	$1.44\times 10^{1}$	8.31	$2.42\times 10^{1}$	5.49	1.10	$5.12\times 10^{-1}$	2.01	$5.13\times 10^{-1}$	$1.80\times 10^{-4}$	$5.87\times 10^{-6}$	$5.09\times 10^{-4}$	$2.06\times 10^{-4}$
	100	$3.04\times 10^{3}$	$1.96\times 10^{3}$	$3.50\times 10^{3}$	$5.65\times 10^{2}$	4.47	3.52	6.26	$9.89\times 10^{-1}$	$4.52\times 10^{-5}$	$1.81\times 10^{-5}$	$9.08\times 10^{-5}$	$2.46\times 10^{-5}$
	200	$4.80\times 10^{4}$	$3.98\times 10^{4}$	$5.98\times 10^{4}$	$6.85\times 10^{3}$	$1.25\times 10^{1}$	$1.12\times 10^{1}$	$1.35\times 10^{1}$	$7.27\times 10^{-1}$	$2.28\times 10^{-4}$	$2.05\times 10^{-5}$	$5.23\times 10^{-4}$	$2.18\times 10^{-4}$
	500	$3.24\times 10^{5}$	$3.16\times 10^{5}$	$3.30\times 10^{5}$	$6.24\times 10^{3}$	$4.89\times 10^{1}$	$4.45\times 10^{1}$	$5.23\times 10^{1}$	2.65	$6.46\times 10^{-4}$	$1.08\times 10^{-4}$	$1.25\times 10^{-3}$	$4.79\times 10^{-4}$
	1000	$1.01\times 10^{6}$	$9.75\times 10^{5}$	$1.07\times 10^{6}$	$3.42\times 10^{4}$	$1.27\times 10^{2}$	$1.22\times 10^{2}$	$1.31\times 10^{2}$	3.64	$2.59\times 10^{-3}$	$3.34\times 10^{-5}$	$5.59\times 10^{-3}$	$2.16\times 10^{-3}$
F7	50	$1.82\times 10^{-1}$	$1.66\times 10^{-1}$	$2.13\times 10^{-1}$	$1.68\times 10^{-2}$	$1.94\times 10^{-3}$	$4.96\times 10^{-4}$	$4.81\times 10^{-3}$	$1.60\times 10^{-3}$	$3.66\times 10^{-5}$	$9.47\times 10^{-6}$	$5.53\times 10^{-5}$	$1.80\times 10^{-5}$
	100	4.65	1.75	8.58	2.42	$2.16\times 10^{-3}$	$9.59\times 10^{-4}$	$3.69\times 10^{-3}$	$8.86\times 10^{-4}$	$5.71\times 10^{-5}$	$4.17\times 10^{-5}$	$8.90\times 10^{-5}$	$1.78\times 10^{-5}$
	200	$1.51\times 10^{2}$	$1.23\times 10^{2}$	$1.68\times 10^{2}$	$1.56\times 10^{1}$	$1.27\times 10^{-3}$	$4.17\times 10^{-4}$	$2.46\times 10^{-3}$	$7.70\times 10^{-4}$	$1.26\times 10^{-4}$	$1.28\times 10^{-5}$	$2.46\times 10^{-4}$	$9.17\times 10^{-5}$
	500	$5.26\times 10^{3}$	$4.66\times 10^{3}$	$5.85\times 10^{3}$	$4.08\times 10^{2}$	$3.81\times 10^{-3}$	$1.78\times 10^{-3}$	$5.06\times 10^{-3}$	$1.17\times 10^{-3}$	$3.05\times 10^{-4}$	$2.18\times 10^{-5}$	$8.95\times 10^{-4}$	$3.16\times 10^{-4}$
	1000	$3.57\times 10^{4}$	$3.11\times 10^{4}$	$3.91\times 10^{4}$	$3.26\times 10^{3}$	$2.75\times 10^{-3}$	$1.28\times 10^{-3}$	$4.36\times 10^{-3}$	$1.21\times 10^{-3}$	$1.44\times 10^{-4}$	$4.37\times 10^{-5}$	$5.09\times 10^{-4}$	$1.82\times 10^{-4}$

**Table A5:** Comparison of optimization results under CEC17 test functions, dimension = 30, maximum iterations = 500.

F	ESOA		PSO [16]		GA [5]		DE [6]		GWO [18]		HHO [83]	
	ave	std	ave	std	ave	std	ave	std	ave	std	ave	std
1	$1.11\times 10^{9}$	$2.07\times 10^{8}$	$5.80\times 10^{11}$	$1.41\times 10^{11}$	$1.50\times 10^{7}$	$2.10\times 10^{7}$	$6.51\times 10^{6}$	$3.90\times 10^{6}$	$2.20\times 10^{10}$	$1.51\times 10^{10}$	$4.25\times 10^{11}$	$1.31\times 10^{10}$
2	inf	inf	inf	inf	inf	inf	inf	inf	inf	inf	inf	inf
3	$3.72\times 10^{4}$	$6.90\times 10^{3}$	$8.35\times 10^{4}$	$2.45\times 10^{4}$	$1.58\times 10^{5}$	$4.99\times 10^{4}$	$1.20\times 10^{5}$	$8.52\times 10^{3}$	$5.03\times 10^{4}$	$9.53\times 10^{3}$	$8.62\times 10^{4}$	$5.54\times 10^{3}$
4	$5.15\times 10^{2}$	$1.79\times 10^{1}$	$5.63\times 10^{3}$	$3.47\times 10^{3}$	$5.83\times 10^{2}$	$3.98\times 10^{1}$	$5.46\times 10^{2}$	7.83	$6.19\times 10^{2}$	$9.33\times 10^{1}$	$9.69\times 10^{3}$	$1.90\times 10^{3}$
5	$6.24\times 10^{2}$	$1.11\times 10^{1}$	$8.06\times 10^{2}$	$3.65\times 10^{1}$	$6.28\times 10^{2}$	$3.11\times 10^{1}$	$6.82\times 10^{2}$	$1.71\times 10^{1}$	$6.22\times 10^{2}$	$4.49\times 10^{1}$	$8.57\times 10^{2}$	$2.66\times 10^{1}$
6	$6.42\times 10^{2}$	6.02	$6.80\times 10^{2}$	4.24	$6.42\times 10^{2}$	5.96	$6.12\times 10^{2}$	1.47	$6.24\times 10^{2}$	7.32	$6.91\times 10^{2}$	8.59
7	$9.63\times 10^{2}$	$2.03\times 10^{1}$	$1.97\times 10^{3}$	$4.73\times 10^{2}$	$1.04\times 10^{3}$	$9.48\times 10^{1}$	$9.25\times 10^{2}$	$1.72\times 10^{1}$	$9.75\times 10^{2}$	$2.10\times 10^{1}$	$1.35\times 10^{3}$	$1.68\times 10^{1}$
8	$9.42\times 10^{2}$	$1.47\times 10^{1}$	$1.12\times 10^{3}$	$2.79\times 10^{1}$	$9.22\times 10^{2}$	$3.39\times 10^{1}$	$9.91\times 10^{2}$	8.15	$9.21\times 10^{2}$	$6.61\times 10^{1}$	$1.10\times 10^{3}$	$2.01\times 10^{1}$
9	$3.42\times 10^{3}$	$1.02\times 10^{3}$	$8.88\times 10^{3}$	$2.05\times 10^{3}$	$2.87\times 10^{3}$	$7.05\times 10^{2}$	$1.06\times 10^{3}$	$6.84\times 10^{1}$	$3.22\times 10^{3}$	$1.12\times 10^{3}$	$6.68\times 10^{3}$	$6.98\times 10^{2}$
10	$5.01\times 10^{3}$	$2.31\times 10^{2}$	$6.07\times 10^{3}$	$6.30\times 10^{2}$	$4.39\times 10^{3}$	$6.59\times 10^{2}$	$7.95\times 10^{3}$	$2.48\times 10^{2}$	$5.75\times 10^{3}$	$1.88\times 10^{3}$	$7.75\times 10^{3}$	$2.53\times 10^{2}$
11	$1.36\times 10^{3}$	$2.26\times 10^{1}$	$2.03\times 10^{3}$	$3.40\times 10^{2}$	$8.53\times 10^{3}$	$7.78\times 10^{3}$	$1.45\times 10^{3}$	$4.09\times 10^{1}$	$1.80\times 10^{3}$	$8.27\times 10^{2}$	$9.61\times 10^{3}$	$1.65\times 10^{3}$
12	$3.65\times 10^{7}$	$9.99\times 10^{6}$	$1.98\times 10^{10}$	$7.39\times 10^{9}$	$1.08\times 10^{7}$	$6.31\times 10^{6}$	$3.99\times 10^{7}$	$1.41\times 10^{7}$	$9.09\times 10^{8}$	$1.15\times 10^{9}$	$4.28\times 10^{10}$	$2.13\times 10^{10}$
13	$3.72\times 10^{6}$	$9.00\times 10^{5}$	$3.51\times 10^{10}$	$2.95\times 10^{10}$	$1.89\times 10^{5}$	$3.41\times 10^{5}$	$1.16\times 10^{7}$	$9.35\times 10^{6}$	$3.76\times 10^{6}$	$4.83\times 10^{6}$	$1.85\times 10^{10}$	$1.22\times 10^{10}$
14	$4.34\times 10^{4}$	$4.59\times 10^{4}$	$7.38\times 10^{4}$	$4.67\times 10^{4}$	$1.31\times 10^{6}$	$1.02\times 10^{6}$	$1.73\times 10^{5}$	$4.02\times 10^{4}$	$9.28\times 10^{5}$	$1.29\times 10^{6}$	$6.44\times 10^{6}$	$4.64\times 10^{6}$
15	$2.19\times 10^{5}$	$1.13\times 10^{5}$	$1.05\times 10^{10}$	$6.13\times 10^{9}$	$1.59\times 10^{4}$	$9.15\times 10^{3}$	$9.86\times 10^{5}$	$4.10\times 10^{5}$	$2.18\times 10^{5}$	$2.24\times 10^{5}$	$9.19\times 10^{8}$	$1.71\times 10^{9}$
16	$2.59\times 10^{3}$	$9.49\times 10^{1}$	$3.76\times 10^{3}$	$5.73\times 10^{2}$	$3.01\times 10^{3}$	$3.48\times 10^{2}$	$3.16\times 10^{3}$	$1.42\times 10^{2}$	$2.47\times 10^{3}$	$3.26\times 10^{2}$	$5.35\times 10^{3}$	$9.24\times 10^{2}$
17	inf	inf	inf	inf	inf	inf	inf	inf	inf	inf	inf	inf
18	$1.88\times 10^{5}$	$6.05\times 10^{4}$	$4.02\times 10^{6}$	$6.21\times 10^{6}$	$4.40\times 10^{6}$	$3.37\times 10^{6}$	$2.00\times 10^{6}$	$4.06\times 10^{5}$	$1.32\times 10^{6}$	$1.11\times 10^{6}$	$2.64\times 10^{7}$	$2.31\times 10^{7}$
19	$1.21\times 10^{6}$	$5.37\times 10^{5}$	$3.96\times 10^{9}$	$6.97\times 10^{9}$	$2.81\times 10^{3}$	$7.51\times 10^{2}$	$1.90\times 10^{6}$	$1.01\times 10^{6}$	$1.69\times 10^{6}$	$1.64\times 10^{6}$	$1.06\times 10^{9}$	$8.97\times 10^{8}$
20	inf	inf	inf	inf	inf	inf	inf	inf	inf	inf	inf	inf
21	$2.42\times 10^{3}$	$1.13\times 10^{1}$	$2.57\times 10^{3}$	$6.65\times 10^{1}$	$2.42\times 10^{3}$	$4.06\times 10^{1}$	$2.48\times 10^{3}$	$1.01\times 10^{1}$	$2.40\times 10^{3}$	$3.21\times 10^{1}$	$2.67\times 10^{3}$	$2.29\times 10^{1}$
22	$2.34\times 10^{3}$	4.65	$7.28\times 10^{3}$	$7.83\times 10^{2}$	$3.99\times 10^{3}$	$2.10\times 10^{3}$	$3.12\times 10^{3}$	$7.51\times 10^{2}$	$6.66\times 10^{3}$	$1.64\times 10^{3}$	$8.69\times 10^{3}$	$1.06\times 10^{3}$
23	$2.81\times 10^{3}$	$1.30\times 10^{1}$	$3.05\times 10^{3}$	$1.08\times 10^{2}$	$2.80\times 10^{3}$	$2.66\times 10^{1}$	$2.83\times 10^{3}$	8.08	$2.80\times 10^{3}$	$6.46\times 10^{1}$	$3.40\times 10^{3}$	$9.57\times 10^{1}$
24	$2.97\times 10^{3}$	$1.96\times 10^{1}$	$3.19\times 10^{3}$	$4.96\times 10^{1}$	$2.98\times 10^{3}$	$3.35\times 10^{1}$	$3.02\times 10^{3}$	7.32	$2.99\times 10^{3}$	$5.33\times 10^{1}$	$3.47\times 10^{3}$	$1.42\times 10^{2}$
25	$2.92\times 10^{3}$	$1.61\times 10^{1}$	$5.81\times 10^{3}$	$1.71\times 10^{3}$	$2.93\times 10^{3}$	$3.34\times 10^{1}$	$2.89\times 10^{3}$	3.17	$2.98\times 10^{3}$	$2.69\times 10^{1}$	$4.30\times 10^{3}$	$4.33\times 10^{2}$
26	$3.10\times 10^{3}$	$6.47\times 10^{1}$	$8.42\times 10^{3}$	$7.39\times 10^{2}$	$5.72\times 10^{3}$	$3.13\times 10^{2}$	$5.48\times 10^{3}$	$1.40\times 10^{2}$	$4.66\times 10^{3}$	$2.34\times 10^{2}$	$1.06\times 10^{4}$	$1.17\times 10^{3}$
27	$3.23\times 10^{3}$	$1.15\times 10^{1}$	$3.39\times 10^{3}$	$8.99\times 10^{1}$	$3.25\times 10^{3}$	$1.70\times 10^{1}$	$3.26\times 10^{3}$	7.45	$3.24\times 10^{3}$	9.68	$3.99\times 10^{3}$	$2.17\times 10^{2}$
28	$3.31\times 10^{3}$	$1.09\times 10^{1}$	$6.75\times 10^{3}$	$1.12\times 10^{3}$	$3.31\times 10^{3}$	$2.93\times 10^{1}$	$3.29\times 10^{3}$	9.66	$3.46\times 10^{3}$	$6.61\times 10^{1}$	$6.21\times 10^{3}$	$3.03\times 10^{2}$
29	inf	inf	inf	inf	inf	inf	inf	inf	inf	inf	inf	inf

**Table A6:** The Wilcoxon Test Result Between ESOA And Other Algorithm On CEC05 Benchmark Functions.

		ESOA vs. PSO	ESOA vs. GA	ESOA vs. DE	ESOA vs. GWO	ESOA vs. HHO
F1	30	$1.25\times 10^{-83}$	$1.08\times 10^{-83}$	$1.25\times 10^{-83}$	$1.26\times 10^{-83}$	$1.24\times 10^{-78}$
	50	$1.25\times 10^{-83}$	$1.19\times 10^{-83}$	$1.24\times 10^{-83}$	$1.26\times 10^{-83}$	$7.18\times 10^{-84}$
	100	$1.25\times 10^{-83}$	$1.19\times 10^{-83}$	$1.19\times 10^{-83}$	$1.26\times 10^{-83}$	$1.79\times 10^{-72}$
	200	$1.26\times 10^{-83}$	$1.20\times 10^{-83}$	$1.17\times 10^{-83}$	$1.26\times 10^{-83}$	$1.14\times 10^{-83}$
	500	$1.26\times 10^{-83}$	$1.23\times 10^{-83}$	$1.11\times 10^{-83}$	$1.26\times 10^{-83}$	$2.46\times 10^{-71}$
	1000	$1.26\times 10^{-83}$	$1.18\times 10^{-83}$	$1.14\times 10^{-83}$	$1.26\times 10^{-83}$	$7.92\times 10^{-80}$
F2	30	$1.24\times 10^{-83}$	$5.74\times 10^{-84}$	$1.24\times 10^{-83}$	$1.26\times 10^{-83}$	$3.95\times 10^{-81}$
	50	$1.21\times 10^{-83}$	$1.16\times 10^{-83}$	$1.20\times 10^{-83}$	$1.26\times 10^{-83}$	$3.95\times 10^{-76}$
	100	$1.17\times 10^{-83}$	$1.18\times 10^{-83}$	$9.69\times 10^{-84}$	$1.26\times 10^{-83}$	$1.17\times 10^{-83}$
	200	$1.17\times 10^{-83}$	$1.19\times 10^{-83}$	$9.72\times 10^{-84}$	$1.26\times 10^{-83}$	$1.17\times 10^{-83}$
	500	N/A	N/A	N/A	N/A	N/A
	1000	N/A	N/A	N/A	N/A	N/A
F3	30	$1.25\times 10^{-83}$	$9.83\times 10^{-84}$	$4.34\times 10^{-84}$	$1.26\times 10^{-83}$	$9.33\times 10^{-84}$
	50	$1.25\times 10^{-83}$	$4.89\times 10^{-84}$	$1.60\times 10^{-85}$	$1.26\times 10^{-83}$	$1.17\times 10^{-83}$
	100	$1.25\times 10^{-83}$	$9.60\times 10^{-84}$	$1.22\times 10^{-84}$	$1.24\times 10^{-83}$	$1.22\times 10^{-83}$
	200	$1.24\times 10^{-83}$	$8.98\times 10^{-84}$	$7.49\times 10^{-87}$	$1.20\times 10^{-83}$	$1.20\times 10^{-83}$
	500	$1.25\times 10^{-83}$	$1.12\times 10^{-83}$	$3.23\times 10^{-93}$	$1.21\times 10^{-83}$	$1.18\times 10^{-83}$
	1000	$1.24\times 10^{-83}$	$9.83\times 10^{-84}$	$2.23\times 10^{-86}$	$1.12\times 10^{-83}$	$1.20\times 10^{-83}$
F4	30	$1.25\times 10^{-83}$	$2.89\times 10^{-84}$	$7.11\times 10^{-84}$	$1.26\times 10^{-83}$	$1.03\times 10^{-83}$
	50	$1.25\times 10^{-83}$	$4.69\times 10^{-84}$	0.00	$1.26\times 10^{-83}$	$1.01\times 10^{-83}$
	100	$1.25\times 10^{-83}$	$5.11\times 10^{-84}$	0.00	$1.26\times 10^{-83}$	$1.88\times 10^{-77}$
	200	$1.24\times 10^{-83}$	$3.31\times 10^{-84}$	0.00	$1.26\times 10^{-83}$	$9.45\times 10^{-84}$
	500	$1.24\times 10^{-83}$	$6.96\times 10^{-84}$	0.00	$1.23\times 10^{-83}$	$1.73\times 10^{-82}$
	1000	$1.24\times 10^{-83}$	$2.32\times 10^{-84}$	0.00	$1.21\times 10^{-83}$	$3.58\times 10^{-68}$
F5	30	$1.24\times 10^{-83}$	$1.25\times 10^{-83}$	$1.25\times 10^{-83}$	$3.32\times 10^{-45}$	$5.66\times 10^{-76}$
	50	$1.25\times 10^{-83}$	$1.25\times 10^{-83}$	$1.23\times 10^{-83}$	$1.70\times 10^{-42}$	$1.43\times 10^{-73}$
	100	$1.26\times 10^{-83}$	$1.25\times 10^{-83}$	$1.24\times 10^{-83}$	$2.07\times 10^{-32}$	$2.28\times 10^{-72}$
	200	$1.26\times 10^{-83}$	$1.25\times 10^{-83}$	$1.24\times 10^{-83}$	$4.61\times 10^{-27}$	$1.46\times 10^{-73}$
	500	$1.25\times 10^{-83}$	$1.25\times 10^{-83}$	$1.22\times 10^{-83}$	$1.23\times 10^{-21}$	$3.57\times 10^{-71}$
	1000	$1.23\times 10^{-83}$	$1.25\times 10^{-83}$	$1.24\times 10^{-83}$	$1.58\times 10^{-20}$	$4.94\times 10^{-81}$
F6	30	$1.09\times 10^{-5}$	$1.11\times 10^{-83}$	$1.32\times 10^{-35}$	$6.84\times 10^{-57}$	$1.90\times 10^{-71}$
	50	$3.27\times 10^{-37}$	$8.32\times 10^{-84}$	$1.24\times 10^{-83}$	$1.16\times 10^{-52}$	$2.15\times 10^{-75}$
	100	$4.56\times 10^{-70}$	$1.14\times 10^{-83}$	$1.22\times 10^{-83}$	$1.21\times 10^{-48}$	$1.28\times 10^{-81}$
	200	$1.26\times 10^{-83}$	$1.23\times 10^{-83}$	$1.19\times 10^{-83}$	$7.08\times 10^{-45}$	$1.31\times 10^{-76}$
	500	$1.26\times 10^{-83}$	$1.24\times 10^{-83}$	$1.15\times 10^{-83}$	$2.79\times 10^{-42}$	$1.84\times 10^{-80}$
	1000	$1.26\times 10^{-83}$	$1.19\times 10^{-83}$	$1.05\times 10^{-83}$	$9.72\times 10^{-41}$	$6.36\times 10^{-78}$
F7	30	$1.18\times 10^{-83}$	$1.26\times 10^{-83}$	$5.19\times 10^{-84}$	$5.01\times 10^{-84}$	$5.62\times 10^{-48}$
	50	$1.20\times 10^{-83}$	$1.26\times 10^{-83}$	$7.70\times 10^{-84}$	$4.89\times 10^{-84}$	$1.76\times 10^{-84}$
	100	$1.06\times 10^{-83}$	$1.26\times 10^{-83}$	$1.15\times 10^{-83}$	$5.07\times 10^{-86}$	$2.27\times 10^{-85}$
	200	$8.37\times 10^{-84}$	$1.26\times 10^{-83}$	$1.16\times 10^{-83}$	$2.79\times 10^{-85}$	$2.21\times 10^{-84}$
	500	$7.59\times 10^{-84}$	$1.26\times 10^{-83}$	$1.12\times 10^{-83}$	$4.72\times 10^{-84}$	$6.32\times 10^{-85}$
	1000	$6.86\times 10^{-95}$	$1.26\times 10^{-83}$	$1.05\times 10^{-83}$	$4.75\times 10^{-84}$	$1.44\times 10^{-81}$

**Table A7:** The Wilcoxon Test Result Between ESOA And Other Algorithm On CEC17 Benchmark Functions.

		ESOA vs. PSO	ESOA vs. GA	ESOA vs. DE	ESOA vs. GWO	ESOA vs. HHO
$F_{1}$	30	$1.15\times 10^{-83}$	$1.49\times 10^{-64}$	$7.73\times 10^{-47}$	$7.59\times 10^{-14}$	$1.26\times 10^{-83}$
$F_{2}$	30	N/A	N/A	N/A	N/A	N/A
$F_{3}$	30	$4.87\times 10^{-81}$	$1.26\times 10^{-83}$	$1.12\times 10^{-83}$	$2.50\times 10^{-82}$	$1.26\times 10^{-83}$
$F_{4}$	30	$1.26\times 10^{-83}$	$5.97\times 10^{-4}$	$1.44\times 10^{-26}$	$1.81\times 10^{-57}$	$1.26\times 10^{-83}$
$F_{5}$	30	$1.26\times 10^{-83}$	$3.35\times 10^{-40}$	$2.64\times 10^{-83}$	$7.09\times 10^{-10}$	$1.26\times 10^{-83}$
$F_{6}$	30	$1.26\times 10^{-83}$	$2.73\times 10^{-71}$	$3.23\times 10^{-50}$	$1.44\times 10^{-23}$	$1.26\times 10^{-83}$
$F_{7}$	30	$1.26\times 10^{-83}$	$1.26\times 10^{-83}$	$1.63\times 10^{-30}$	$2.45\times 10^{-13}$	$1.26\times 10^{-83}$
$F_{8}$	30	$1.03\times 10^{-83}$	$2.68\times 10^{-80}$	$6.81\times 10^{-11}$	$3.17\times 10^{-22}$	$2.62\times 10^{-83}$
$F_{9}$	30	$3.82\times 10^{-82}$	$2.80\times 10^{-74}$	$4.34\times 10^{-51}$	$8.96\times 10^{-80}$	$9.99\times 10^{-56}$
$F_{10}$	30	$3.69\times 10^{-45}$	$1.87\times 10^{-83}$	$7.29\times 10^{-84}$	$1.93\times 10^{-81}$	$1.33\times 10^{-83}$
$F_{11}$	30	$2.74\times 10^{-82}$	$1.26\times 10^{-83}$	$2.13\times 10^{-54}$	$4.73\times 10^{-72}$	$1.25\times 10^{-83}$
$F_{12}$	30	$1.26\times 10^{-83}$	$3.69\times 10^{-35}$	$1.16\times 10^{-6}$	$2.29\times 10^{-62}$	$1.26\times 10^{-83}$
$F_{13}$	30	$1.27\times 10^{-83}$	$3.06\times 10^{-22}$	$6.17\times 10^{-33}$	$3.57\times 10^{-46}$	$1.26\times 10^{-83}$
$F_{14}$	30	$8.57\times 10^{-66}$	$1.87\times 10^{-63}$	$1.90\times 10^{-37}$	$1.18\times 10^{-83}$	$1.26\times 10^{-83}$
$F_{15}$	30	$1.97\times 10^{-46}$	$1.49\times 10^{-20}$	$1.20\times 10^{-83}$	$5.85\times 10^{-73}$	$1.26\times 10^{-83}$
$F_{16}$	30	$2.53\times 10^{-76}$	$1.08\times 10^{-1}$	$1.74\times 10^{-76}$	$1.21\times 10^{-82}$	$2.87\times 10^{-76}$
$F_{17}$	30	N/A	N/A	N/A	N/A	N/A
$F_{18}$	30	$2.76\times 10^{-14}$	$1.26\times 10^{-83}$	$9.89\times 10^{-84}$	$1.94\times 10^{-17}$	$1.26\times 10^{-83}$
$F_{19}$	30	$3.27\times 10^{-18}$	$4.91\times 10^{-13}$	$2.83\times 10^{-37}$	$1.15\times 10^{-72}$	$1.26\times 10^{-83}$
$F_{20}$	30	N/A	N/A	N/A	N/A	N/A
$F_{21}$	30	$3.91\times 10^{-79}$	$7.58\times 10^{-74}$	$2.81\times 10^{-62}$	$3.86\times 10^{-6}$	$1.26\times 10^{-83}$
$F_{22}$	30	$1.26\times 10^{-83}$	$1.46\times 10^{-4}$	$2.08\times 10^{-81}$	$1.24\times 10^{-83}$	$4.64\times 10^{-83}$
$F_{23}$	30	$1.21\times 10^{-83}$	$3.94\times 10^{-82}$	$2.41\times 10^{-20}$	$1.46\times 10^{-75}$	$1.49\times 10^{-83}$
$F_{24}$	30	$3.27\times 10^{-54}$	$7.40\times 10^{-79}$	$8.32\times 10^{-19}$	$2.84\times 10^{-30}$	$1.45\times 10^{-83}$
$F_{25}$	30	$1.26\times 10^{-83}$	$3.93\times 10^{-6}$	$2.23\times 10^{-17}$	$1.76\times 10^{-4}$	$1.26\times 10^{-83}$
$F_{26}$	30	$1.32\times 10^{-83}$	$1.63\times 10^{-54}$	$1.11\times 10^{-72}$	$1.33\times 10^{-44}$	$1.26\times 10^{-83}$
$F_{27}$	30	$5.37\times 10^{-67}$	$1.81\times 10^{-75}$	$4.86\times 10^{-79}$	$1.97\times 10^{-4}$	$1.26\times 10^{-83}$
$F_{28}$	30	$1.25\times 10^{-83}$	$1.26\times 10^{-83}$	$6.87\times 10^{-10}$	$6.62\times 10^{-4}$	$1.37\times 10^{-83}$
$F_{29}$	30	N/A	N/A	N/A	N/A	N/A
